# Design, synthesis, and antimicrobial evaluation of novel quinazoline piperazine phosphorodiamidate hybrids as potent DNA gyrase inhibitors

**DOI:** 10.1038/s41598-025-11516-7

**Published:** 2025-09-30

**Authors:** Suresh Babu Donka, Sajitha Kethineni, Bala Yesu Valaparla, Anusha Bheemreddy, Manjunadh D. Meti, Uttam A. More, Venkata Subbaiah Kotakadi, Murali Vatturu, Srinivasulu Doddaga

**Affiliations:** 1 Department of Chemistry, Sri Venkateswara University, Tirupati, 517502 Andhra Pradesh India; 2 Department of Chemistry, SGK Government Degree College, Vinukonda, Palanadu, 522647 Andhra Pradesh India; 3 Department of Chemistry, Silver Jubilee Government College, Cluster University, Kurnool, 518502 Andhra Pradesh India; 4 Department of Plant Sciences, School of Life Sciences, University of Hyderabad, Gachibowli, 500046 Telangana India; 5 Department of Pharmaceutical Chemistry, Shree Dhanvantary Pharmacy College, Kim, Suram, 394110 India; 6 DST PURSE centre, Sri Venkateswara University, Tirupati, 517502 Andhra Pradesh India

**Keywords:** Quinazoline, Morpholine, Piperazine, Phosphorodiamidate, Anti-microbial activity, *In Silico* studies, Chemical modification, Biochemistry, Chemical biology, Drug discovery

## Abstract

Antimicrobial resistance poses a critical challenge to global public health, exacerbating morbidity and mortality associated with bacterial infections. This study addresses the urgent need for novel antibacterial agents by exploring the design and synthesis of quinazoline-piperazine phosphorodiamidate hybrids (**6a-g)** as potential DNA gyrase inhibitors. Antibacterial activity was evaluated using the agar well diffusion method, revealing significant inhibition zones for compounds **6f**,** 6 g**,** 6a**, and **6c** compared to the standard drug Amoxyclav. Minimum inhibitory concentration (MIC) measurements further supported the potent antibacterial effects of these compounds. Additionally, compounds **6f**,** 6 g**, and **6a** exhibited notable antifungal activity superior to Fluconazole. Molecular docking simulations against DNA gyrase demonstrated strong binding affinities of compounds **6f** and **6a** with dock scores surpassing that of a standard antibiotic, ciprofloxacin. Detailed analysis of binding interactions highlighted key residues involved in stabilizing the ligand-protein complexes, providing insights into their mechanism of action. Furthermore, in silico ADMET prediction studies revealed that the targeted analogs satisfied the drug like characteristics of CNS acting drugs against antimicrobial diseases.

**Supplementary Information:**

The online version contains supplementary material available at 10.1038/s41598-025-11516-7.

## Introduction

 Antimicrobial drugs, including antibiotics, antivirals, and antimalarials, have been cornerstone innovations in modern medicine, dramatically improving public health outcomes. However, the rise of antimicrobial resistance (AMR) has emerged as a major global health crisis, threatening the continued effectiveness of these essential treatments. In 2019 alone, AMR was directly responsible for 1.27 million deaths and contributed to nearly 5 million deaths worldwide, placing significant pressure on healthcare systems and economies^1^. Among AMR, bacterial pathogens, which affect a wide array of hosts from humans to plants, cause substantial morbidity, mortality, and economic losses, especially in healthcare and agriculture^2^. The excessive and improper use of antibiotics has led to the rapid development of resistant bacterial strains, reducing the effectiveness of existing treatments and underscoring the urgent need for innovative antibacterial agents^1^. Developing new drugs with unique chemical structures and mechanisms of action is essential to counteract resistance. One promising approach is to create small-molecule compounds that focus on processes unique to bacteria, like DNA topoisomerases, which are important for how bacteria copy and fix their DNA but are not found in human cells^3^. This approach offers the potential for selective, effective treatments against resistant pathogens, addressing the growing problem of antibiotic resistance.

Quinazoline derivatives, a key group of nitrogen-based compounds, have become very important in medicinal chemistry because they have many useful effects on health. These compounds are of particular interest for their broad spectrum of biological activities, which encompass antimicrobial^4^, anticancer^5^, antiviral^6^, antiinflammatory^7^, antihypertensive^8^, anticonvulsant^9^, antioxidant^10^, anti-HIV^11^, and antimalarial^12^. The quinazoline scaffold has garnered attention as a “privileged structure” in drug design, owing to its ability to interact with a variety of biological targets, including enzymes, receptors, and kinases. In the context of antimicrobial therapy, quinazoline derivatives have proven their ability to inhibit key bacterial enzymes, such as DNA gyrase and topoisomerases, placing them as potential candidates for novel antibacterial agents, especially in the face of rising antimicrobial resistance. Moreover, quinazoline-based compounds are also recognized for their minimal side effects and excellent pharmacokinetic profiles, making them highly attractive for further development. With a growing body of evidence supporting their efficacy, quinazoline derivatives are poised to play a critical role in the next generation of antimicrobial agents, as well as in the treatment of various other diseases, underscoring their importance in the ongoing quest for innovative therapeutic solutions^13^.

The piperazine moiety, a six-membered nitrogen-containing heterocyclic compound, has become a highly valued structural motif in drug discovery due to its unique physicochemical properties. Piperazine has two nitrogen atoms that face each other, which provides important benefits like a large polar surface area, a strong structure, and the ability to accept and donate hydrogen bonds. These features improve how well the drug binds to its target, how specific it is, and how well it dissolves in water, while also making it easier for the body to absorb and use, which is important for creating effective medicines.Over the years, piperazine and its substituted derivatives have been incorporated into a wide range of marketed pharmaceuticals, demonstrating their versatility across various therapeutic areas, including antibiotics^14^ anticancer agents^14^, antimicrobials^15^, antipsychotics^16^, antiviral agents^17^, antiinflammatory^18^, antimycobacterial^19^, antiparasitic^20^, anticonvulsant^21^, antituberculosis effects^22^, as well as acetylcholinesterase inhibition^23^ and more. Particularly in antimicrobial drug development, the incorporation of piperazine has shown promising results, significantly improving the efficacy of antibacterial agents. Piperazine helps connect different active parts of drugs, like quinazoline or phosphonamidate, which leads to the creation of new molecules that work better against bacteria. This wide range of biological effects highlights the great potential of piperazine-based compounds in finding new drugs, making them an important part of creating new treatments that work against a variety of infections, especially those caused by resistant germs^25^.

**Fig. 1:**
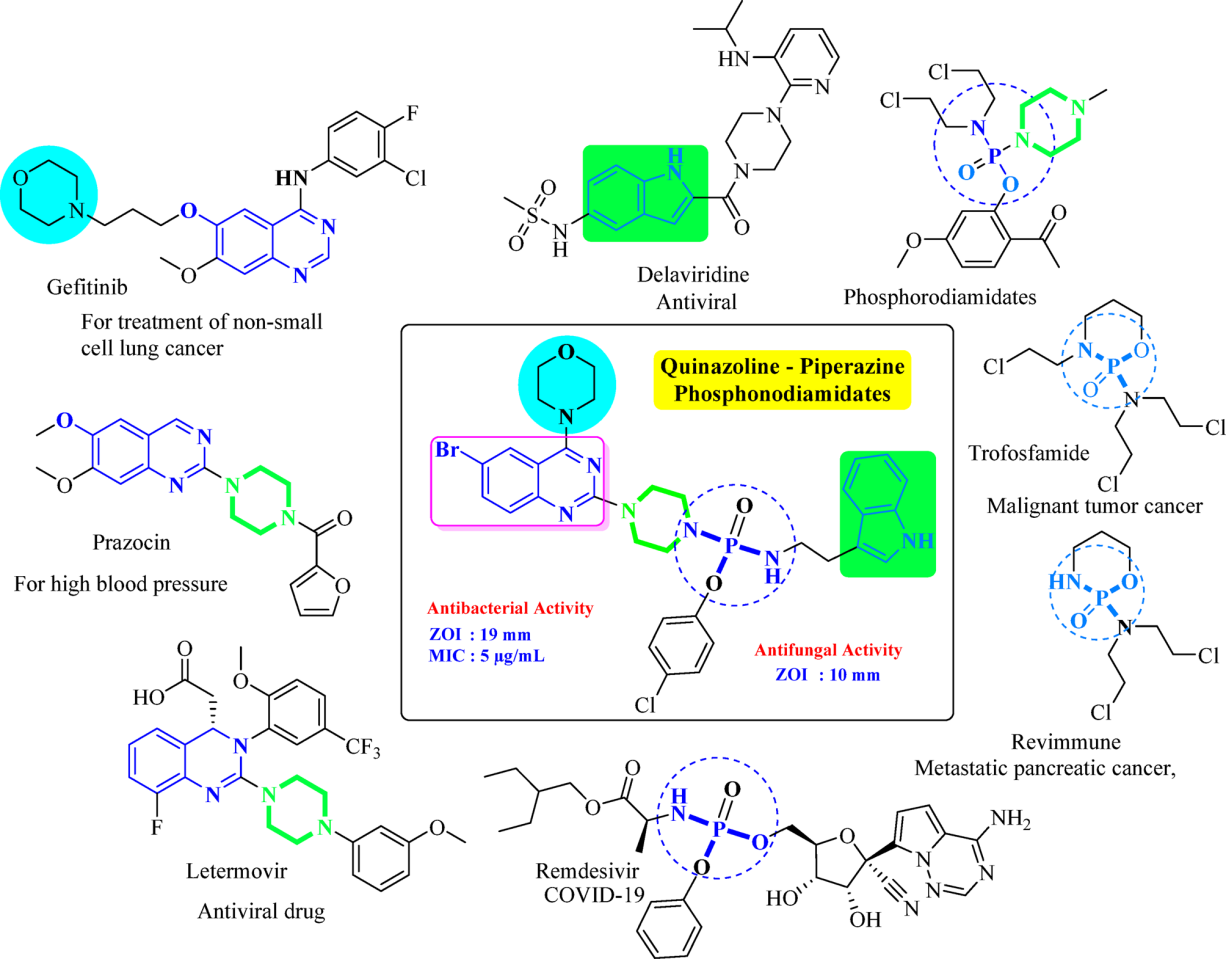
Hybrid compounds combining quinazoline, morpholine, piperazine, and phosphonamidate scaffolds.

Phosphorus is a crucial element in all living organisms, playing a fundamental role in biological processes such as ATP-driven cellular energy transfer and the structural integrity of nucleic acids (DNA and RNA). Its versatile chemical properties have made phosphorus compounds essential in the design of therapeutic agents, particularly organophosphorus derivatives like phosphonamidates and phosphoramidates^26^. These compounds have garnered significant attention due to their broad spectrum of biological activities, including antimicrobial^27^, anticancer^28^, antiviral^29^, and antiparasitic effects^30^.

Phosphonamidates have become promising options for prodrugs, particularly for targeting bacterial enzymes like topoisomerases that are essential for bacterial DNA replication and repair but are not found in human cells. By improving how therapeutic compounds work in the body, phosphonamidates provide a focused way to fight bacterial infections, making them important in the battle against drug-resistant germs for effective treatments of bacterial diseases^31^.

We aimed to synthesize a new type of molecules that include quinazoline, morpholine, piperazine, and phosphorodiamidate structures, based on some well-known compounds. In this study, we suggest making a new group of antibacterial agents by mixing quinazoline, morpholine, piperazine, and phosphonamidate structures into combined compounds. This approach is inspired by the therapeutic potential of well-established molecules like Gefitinib, Parzocin, Letermovir, Remdesivir, Revimmune, Trofosfamide, and Delaviridine, which have demonstrated efficacy in various treatments^32^. By synthesizing quinazoline-based phosphoramidate-piperazine derivatives, we aim to capitalize on the beneficial properties of each component. The quinazoline backbone offers antimicrobial activity, while the morpholine and piperazine parts work together to make the compounds easier to dissolve and more effective in the body. Incorporating phosphorodiamidate functionalities further refines the target specificity and enhances the biological potency of these hybrid molecules (Fig. 1). These compounds will be carefully synthesized, characterized, and evaluated for their antimicrobial properties, with the goal of identifying promising candidates for combating bacterial infections. To better understand the potential of these hybrid compounds, we employed molecular docking studies to examine their interactions with bacterial DNA gyrase, a key enzyme involved in DNA replication and transcription, and also evaluated in silico ADMET prediction to know the drug-like character.

DNA gyrase, a type II topoisomerase, is a vital enzyme that regulates the structural state of DNA by converting it between relaxed and supercoiled forms^32^. It plays a key role in essential cellular processes such as replication, transcription, recombination, repair, and chromosome organization. Due to its critical function in maintaining genomic integrity, DNA gyrase has become a promising target for the development of novel antibacterial agents^33^. By simulating the binding of the novel quinazoline-piperazine phosphorodiamidate hybrids to DNA gyrase, we aim to predict their binding affinity and elucidate the nature of their interactions with the enzyme’s active site.

## Results and discussion

### Chemistry

**Fig. 2:**
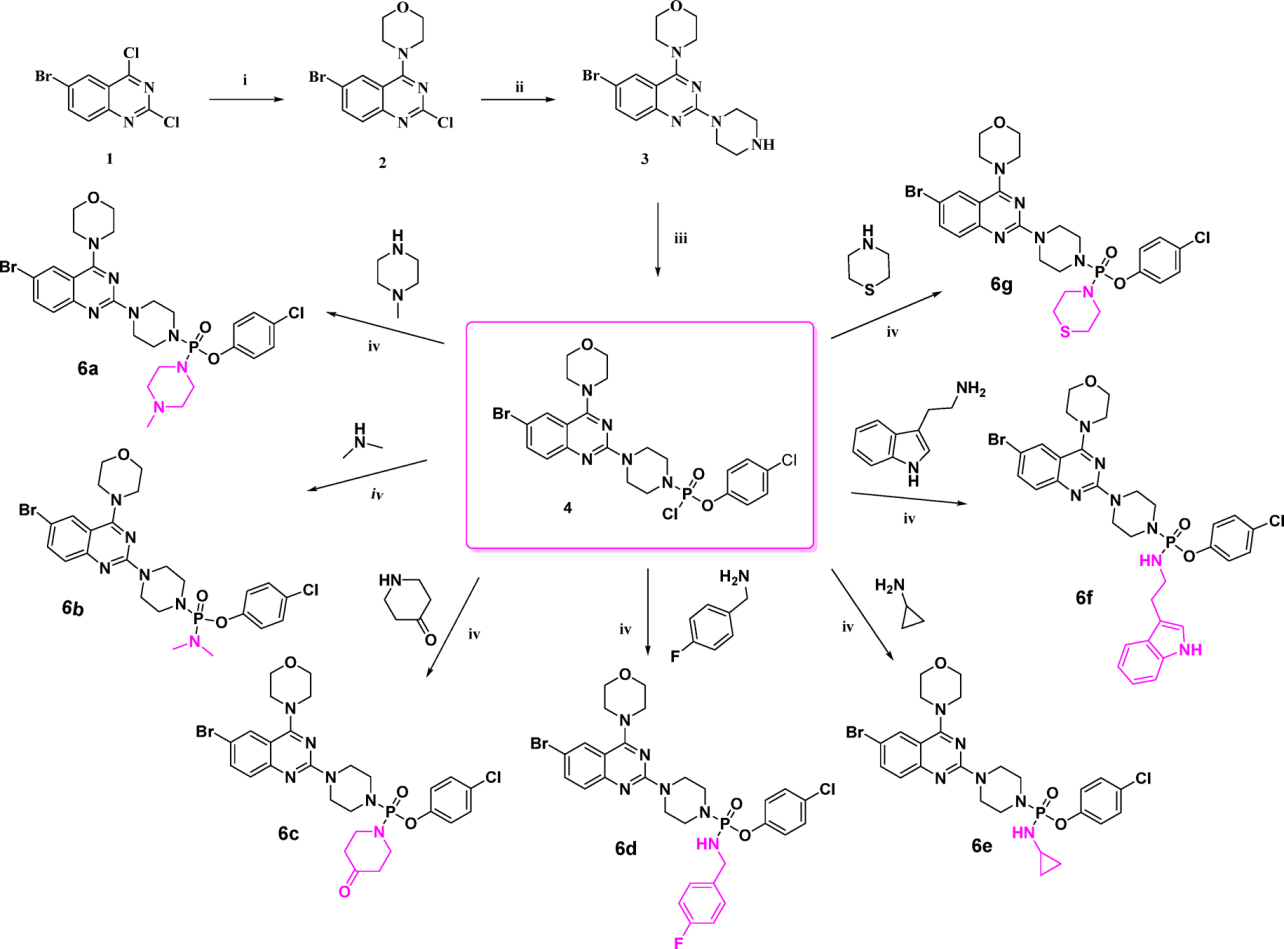
Synthesis of Quinazoline-Phosphonamidate-Piperazine derivatives (**6a-g**). **Characterization**: The structures of the Quinazoline-Phosphonamidate-Piperazine derivatives (**6a-g**) were determined using various spectroscopic methods, including FT-IR, ¹H-NMR, ¹³C-NMR, ^31^P-NMR, ES-MS, and elemental analysis. The FT-IR spectra of compounds **6a-g** display characteristic absorption bands for N-H (3197–3184 cm^−1^), C-H (2856–2848 cm^−1^), C = C (1602–1598 cm^−1^), C = N (1548–1539 cm^−1^), P = O (1219–1209 cm^−1^), P-O-C (1163–1159 cm^−1^) stretchings. In the ¹H-NMR spectra, aromatic protons resonate between δ 8.32–6.94. Oxygen attached methylene protons of morpholine appeared in the downfield region δ 4.20–3.61, while nitrogen attached methylene protons of morpholine appeared in the up field between δ 3.87–3.22. The ¹³C-NMR spectra showed distinct shifts for heteroatom attached quinazoline carbons (δ 165.8–150.0).The carbon signals for aromatic moiety varied with the substituents with the range (δ 155.3-110.6), piperazine and morpholine carbons appeared (δ 66.6–44.3). The ^31^P-NMR spectra showed the presence of phosphorus in the title compounds. Finally, ES-MS and elemental analysis further confirmed the molecular structures, as the m/z values were well corroborated with the expected molecular weights of the target compounds. Reagents and conditions: (i) Morpholine, DCM, 0 °C, 2 h; (ii) Piperazine, K_2_CO_3_, 1,4-dioxane, 110 °C, 2 h; (iii) Dichlorophosphinic acid, 4 chloro-phenyl ester, NEt_3_, THF, 25 °C, 8 h; (iv) 1°/2° amines, NEt_3_, THF, 25 °C, 8 h.

The synthesis of the title compounds (**6a-g**) was carried out in a multi-step process (Fig. 2), starting with the nucleophilic substitution of 6-bromo-2,4-dichloroquinazoline (**1**) with morpholine in DCM at 0 °C to produce 6-Bromo-2-chloro-4-morpholin-4-yl-quinazoline (**2**). This intermediate was then reacted with piperazine and K_2_CO_3_ in 1,4-dioxane under reflux conditions to afford 6-Bromo-4-Morpholin-4-yl-2-Piperazin-1-yl-quinazoline (**3**). In the final step, compound **3** was treated with triethylamine (TEA) and 4-chlorophenyl dichlorophosphinic acid ester in THF under a nitrogen atmosphere, followed by the addition of various substituted primary and secondary amines in situ, yielding a series of quinazoline derivatives (**6a-g**). The synthesized compounds were purified by column chromatography using a 2–5% methanol in chloroform gradient as the eluent. All compounds were characterized by IR, ^1^H-NMR, ^13^C-NMR, ^31^P-NMR, mass spectrometry and elemental analysis, as detailed in the experimental section. Furthermore, the synthesized compounds were evaluated for their antimicrobial activity, showcasing the potential biological significance of these novel quinazoline-based derivatives.

### Antibacterial activity

**Fig. 3:**
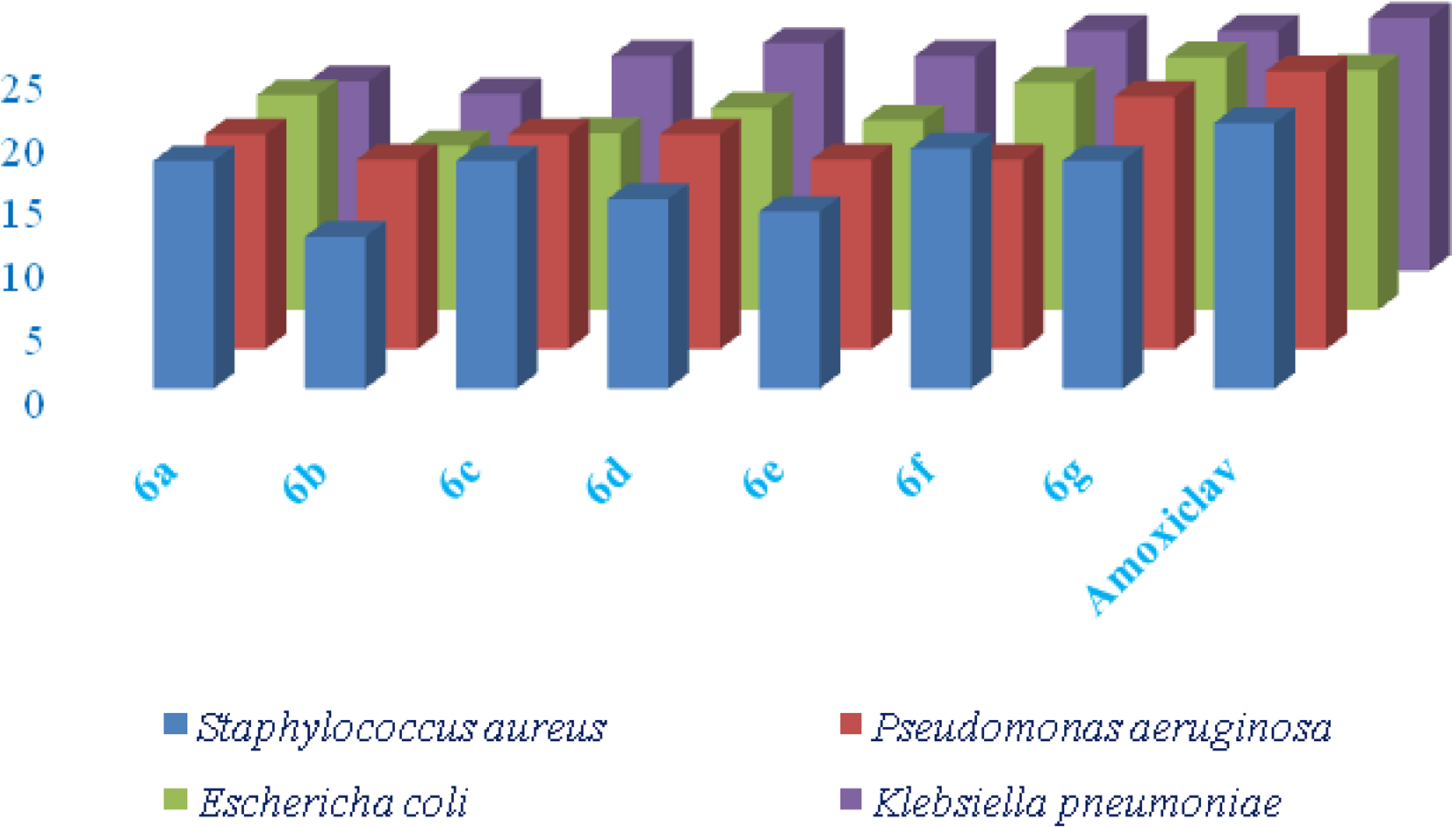
Antibacterial activity of compounds 6a-g (ZOI in mm).

The antibacterial activity of the synthesized compounds **(6a-g)** was assessed through Agar gel well diffusion method and measured the Zone of Inhibition (ZOI) and the Minimum Inhibitory Concentration (MIC) using Mueller Hinton Broth method. These two methods provided valuable insight into the efficacy of the compounds against both Gram-positive (*Staphylococcus aureus* and *Pseudomonas aeruginosa*) and Gram-negative (*Escherichia coli* and *Klebsiella pneumoniae*) bacterial strains. The ZOI and MIC results, as presented in Tables 1 and 2 along with control standard antibiotic, Amoxyclav *Ac* ^*30*^ (SD063, Himedia), revealed that the antibacterial activity of the compounds was influenced by the structure of the substituents attached to the core molecular framework, and the results were also represented in a graphical manner in Fig. 3.

**Table 1:** Antibacterial activity of compounds 6a-g (ZOI in mm).

Gram-positive bacteria Gram-negative bacteria				
Compound	Staphylococcus aureus	Pseudomonas aeruginosa	Escherichia coli	Klebsiella pneumoniae
**6a**	18	17	17	15
**6b**	12	15	13	14
**6c**	18	17	14	17
**6d**	15	17	16	18
**6e**	14	15	15	17
**6f**	19	15	18	19
**6g**	18	20	20	19
**Amoxiclav**	**21**	**22**	**19**	**20**

#### Zone of inhibition (ZOI) analysis

On the other hand, compound **6a** (N-methyl piperazine), while also effective, displayed somewhat lower ZOI values (18 against *Staphylococcus aureus* and 17 against *Pseudomonas aeruginosa*, 17 against *Escherichia coli*) compared to **6f** and **6 g**. The presence of n-methyl piperazine group may influence the compound’s membrane permeability, contributing to its antibacterial action but possibly limiting its potency relative to compounds with more rigid or aromatic structures. Compounds like **6b** (Dimethylamine) and **6e** (cyclopropylamine) showed relatively weaker ZOI values, particularly against Gram-positive bacteria. For instance, compound **6b** showed ZOIs of **12** against *Staphylococcus aureus* and 15 against *Pseudomonas aeruginosa.* These results suggest that less bulky amine groups may reduce antibacterial effectiveness, particularly against Gram-positive strains. Similarly, the **6e** (cyclopropylamine) group may introduce steric hindrance, diminishing its ability to effectively penetrate bacterial membranes.

#### Minimum inhibitory concentration (MIC) analysis

The MIC values, which provide information of the lowest concentration at which bacterial growth is inhibited, further clarified the relative potency of the compounds. The results of MIC values of compound **6 g** were consistently the lowest across all bacterial strains, ranging from 2.5 to 5 µg/mL, indicating its high potency. This correlates well with its highly ZOI values of antibacterial activity of the same sample which suggests that the **6 g** (Thiomorpholine) group significantly contributes to both the inhibition of bacterial growth and the efficiency of the compound at lower concentrations. Compounds **6a**,** 6c**, and **6d** also showed promising MIC values of 2.5 µg/mL against several strains, reflecting their strong antibacterial activity. Compound **6a** containing N-methylpiperazine group and compound **6c** containing piperidone group appear to be beneficial in enhancing activity compound **6c** displaying potent effects against Gram-positive and Gram-negative bacteria (2.5 µg/mL against *Klebsiella pneumoniae* and *Staphylococcus aureus*). Compound **6d** having 4-fluorobenzylamine group, while similar in terms of MIC values (2.5 µg/mL), suggests that the electron-withdrawing fluorine atom may contribute to increase the electron density on the nitrogen atom, which can enhance the compound binding affinity to bacterial targets.

Finally the results revealed and concluded that the most of the synthesized compounds **6a-g**, showed MIC between 2.5 µg/mL to 5 µg/mL (i.e. 2.5 mcg/mL, 5 mcg/mL). Among the synthesized compounds, **6d** and **6 g** have shown potency with MIC of value 2.5 mcg/mL in both gram positive and gram negative bacteria whereas the other compounds have shown similar results with a little higher concentration of 5 mcg/mL as the results are tabulated in Table 2.

**Table 2:** Antibacterial activity of compounds 6a-g (MIC in µg/mL).

Gram-positive bacteria Gram-negative bacteria				
Compound	Staphylococcus aureus	Pseudomonas aeruginosa	Eschericha coli	Klebsiella pneumoniae
**6a**	2.5	5	2.5	2.5
**6b**	5	2.5	5	5
**6c**	2.5	2.5	5	2.5
**6d**	2.5	2.5	2.5	2.5
**6e**	5	2.5	2.5	2.5
**6f**	2.5	5	2.5	2.5
**6 g**	2.5	2.5	2.5	2.5
**Amoxyclav**	2.5	2.5	2.5	2.5

**Fig. 4:**
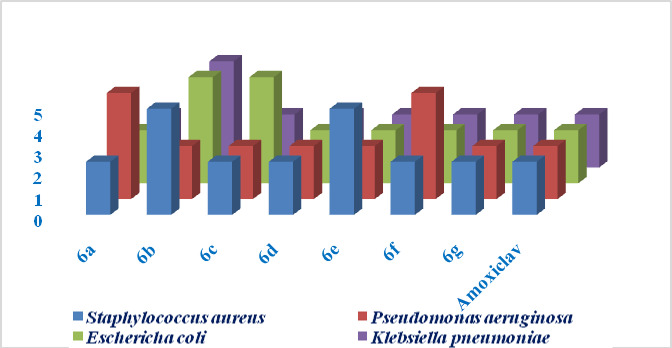
Antibacterial activity of compounds 6a-g (MIC in µg/mL).

Compounds **6b** (dimethylamine) and **6e** (cyclopropylamine) although effective, required higher concentrations to achieve bacterial inhibition (5 µg/mL), highlighting a reduction in potency when compared to the most effective compounds. The reduced efficacy of these compounds could be attributed to the bulkiness or less favorable electronic properties of these substituents, which might hinder optimal interaction with bacterial cell membranes or essential enzymes and represented in Fig. 4.

#### Structure-activity relationship (SAR)

The structure-activity relationship (SAR) analysis reveals that the amine substituent plays a crucial role in determining the antibacterial activity of these compounds. The compounds having thiomorpholine (**6g**) and compound (**6f**) indole-3-ethanamine groups seem to be key contributors to the potent antibacterial effects, particularly due to their ability to enhance membrane permeability and interact with bacterial targets. The rigidity and planarity of the indole and piperidone groups are also advantageous in maintaining favorable interactions with bacterial cell components. On the other hand, more flexible or bulky amines like **6b** (dimethylamine) and **6e** (cyclopropylamine) were less effective, possibly due to hindered membrane penetration or decreased binding affinity to bacterial targets. 4-Fluorobenzylamine in **6d** displayed an interesting balance, where its electron-withdrawing property improved the overall activity against certain strains, especially *Klebsiella pneumoniae*, but it was not as potent as **6g** or **6f**. In assumption, the antibacterial activity of compounds **6a-g** is largely dictated by the chemical structure, particularly the nature of the amine substituent. The most effective compounds, such as **6g** (Thiomorpholine) and **6f** (indole-3-ethanamine) demonstrated broad-spectrum antibacterial activity with low MIC values correlating with their favorable structural characteristics, including cyclic amines and aromatic systems. These findings suggest that compounds with such moieties are strong candidates for further development as antibacterial agents. Conversely, compounds with less activity substituents, such as **6b** (dimethylamine) and **6e** (cyclopropylamine) exhibited lower potency, indicating that strategic modifications to the molecular framework could further enhance antibacterial efficacy. Further optimization of these structures could lead to even more potent antimicrobial agents.

### Antifungal activity

**Fig. 5:**
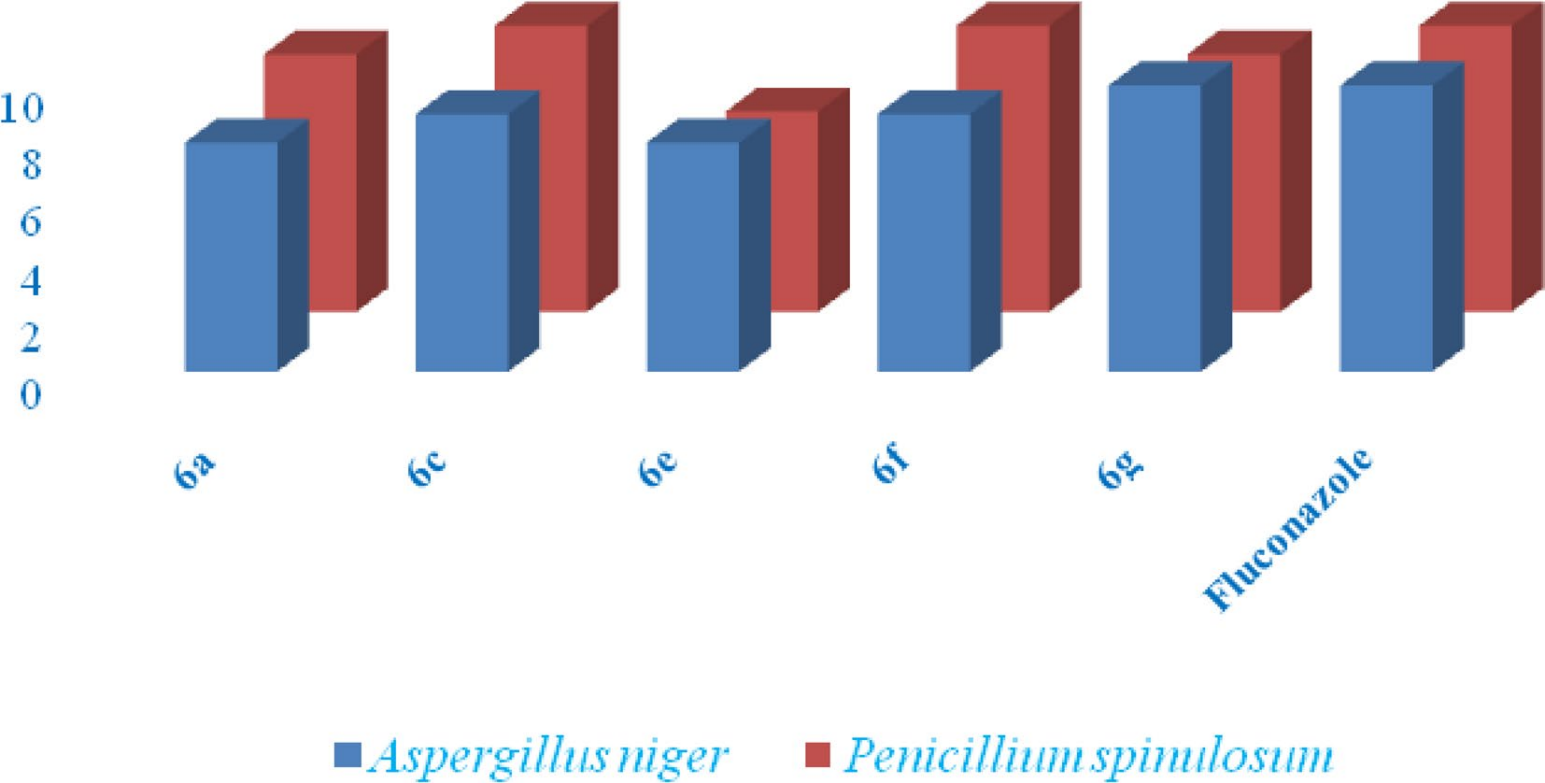
Antifungal activity of compounds 6a, 6c, 6e, 6f and 6 g (MIC in µg/mL).

The antifungal activity of compounds **6a**,** 6c**,** 6e**,** 6f** and **6 g** was assessed against two fungal strains, *Aspergillus niger* and *Penicillium spinulosum*, using the poison plate technique. The results are expressed as Zone of Inhibition (ZOI) in Table 3 and represented in Fig. 5, demonstrated varying degrees of antifungal activity, with compounds showing both moderate and significant efficacy. Compounds **6f** (indole-3-ethanamine), **6 g** (thiomorpholine) and **6c** (piperidone) exhibited the strongest antifungal effects. Compound **6 g** (thiomorpholine) group on the N-P-N bond, showed the highest ZOI of 10 against *Aspergillus niger* and 9 against *Penicillium spinulosum*. The thiomorpholine ring, with its electron-donating properties, likely enhances the compound ability to penetrate fungal membranes and interact with fungal enzymes, thereby improving its antifungal activity. Similarly, **6c** (piperidone), featuring a piperidone group on the N-P-N bond, also showed significant antifungal activity, with a ZOI of 9 against *Aspergillus niger* and 10 against *Penicillium spinulosum*. The piperidone ring is a rigid structure that may increase binding affinity to fungal cell wall components, making it particularly effective against fungal strains. The rigidity and planarity of these rings are crucial for enhancing antifungal activity, as they can better interact with fungal cell membranes and metabolic pathways. On the other hand, Compounds **6a** (N-methylpiperazine) and **6e** (cyclopropylamine) showed moderate antifungal activity, with ZOI values of 8 for both against *Aspergillus niger* and slightly higher values against *peniciliium spinulosum* (9 for **6a** and 7 for **6e**). The N-methylpiperazine group in **6a** and the cyclopropylamine ring in **6e**, while still effective, seem less potent compared to **6 g** and **6c**. These compounds may exhibit some degree of antifungal activity due to their ability to interact with fungal cell membranes, but their less rigid and more flexible structures could hinder optimal membrane penetration and interaction with fungal enzymes. The N-methylpiperazine group in **6a** and the cyclopropylamine ring in **6e** might reduce the overall efficiency of these compounds by limiting the structural rigidity necessary for efficient binding to fungal targets, thus explaining the slightly lower ZOI values observed for these compounds.

**Table 3:** Antifungal activity of tested compounds 6a, 6c, 6e, 6f and 6 g (ZOI in mm).

Compound	Aspergillus niger	Penicillium spinulosum
6a	08	09
6c	09	10
6e	08	07
6f	09	10
6 g	10	09
Fluconazole	10	10

The fluconazole control, with a ZOI of 10 against both fungal strains, provides a benchmark for comparison. Compounds **6c** and **6g** demonstrated antifungal activity comparable to or exceeding that of fluconazole, suggesting their potential as strong antifungal agents. Overall, the antifungal activity of the compounds can be attributed to the nature of the substituents on the N-P-N bond. Compounds with rigid structures like piperidone (**6c**) and thiomorpholine (**6g**) displayed superior activity, likely due to their enhanced ability to penetrate fungal membranes and bind to essential fungal enzymes or cell wall components. In contrast, more flexible groups like **6a** (N-methylpiperazine) and **6e** (cyclopropylamine) exhibited moderate activity, which can be linked to reduced membrane interaction and binding efficiency. These findings highlight the importance of structural features such as rigidity and planarity in optimizing antifungal activity, suggesting that **6c** and **6 g** are promising candidates for further development as antifungal agents.

### Docking analysis

The series of the synthesized compounds **6a-g** showed good antimicrobial activity, which is clearly evident from the in vitro studies. The molecular docking simulations showed that the compounds exhibit reliable binding affinities to the active sites of the selected targets used DNA gyr A (PDB ID: 1AB4). The dock score, hydrogen, and Van der Waal’s bond interactions, as well as other parameters, are used to evaluate the binding activities of.

**Table 4:** Molecular interaction of compounds with DNA gyrase of bacteria binding affinity scores, molecular interaction and bond length.

Compound	Substituents	Bond Length (A^o^)	Binding Affinity (k.cal/mol)	DNA gyrase binding site (residue)
**6a**	N-methyl piperazine	2.93, 3.20, 3.46, 3.23, 2.93, 3.75	-7.30	ASP 87, ASP 87, SER 116, ALA 117, ASP 87, ARG 91
**6b**	Dimethylamine	2.95, 4.94	-6.90	ARG 91, GLY 114
**6c**	Piperidone	3.50, 4.42	-7.12	SER 116, ARG 91
**6d**	4-F-Benzylamine	3.08, 3.36, 3.96, 3.56	-7.23	GLN 94, ALA 117, ASP 87, ASP 87
**6e**	Cyclopropylamine	3.62, 3.73	-6.80	ASP 87, ASP 87
**6f**	Indole-3-ethanamine	3.04, 3.11, 2.97, 3.39	-7.57	GLN 94, ALA 117 ASP 87, ASP 87
**6 g**	Thiomorpholine	4.35	-6.64	GLY 114
Streptomycin		2.72, 2.93, 2.81, 3.17, 2.93, 2.97	-7.38	GLY 114, ASP 87, SER 111, ASP 115, PHE 96, GLN 94
Ciprofloxacin		3.49	-5.10	ARG 91
Tetracycline		3.40, 3.09, 2.86	-5.80	PHE 96, GLN 94, ARG 91
Chloramphenicol		3.01, 3.12	-5.73	ALA 117, ARG 91

synthesized compounds at the active sites with the various amino acid residues, including ARG 91, PHE 96, GLY 114, SER 116, ALA 117, THR 219, GLN 267, and ASN 269 at the active sites of the receptors as shown in Tables 4 and 5, the corresponding 2D and 3D docking images are represented in Table 6.

The ligand-protein complex showed H-bonding, van der waal, and π-H interactions. Among all the synthesized molecules, compounds **6f** and **6a** at the active site of DNA gyrase (PDB ID: 1AB4) have shown a high docked score of −7.57 and − 7.30 k.cal/mol respectively than ciprofloxacin (−5.10 k.cal/mol) showcasing its robust interaction with the target protein. Notably, interactions with residues, such as GLN 94 (3.08 Å), ALA 117 (3.36 Å), ASP 87 (3.96 Å), ASP 87 (3.46 Å), further support the strong binding potential of **6f** along with rmsd_refine value 2.13 suggests a close structural alignment with the crystal structure. Similarly, compounds **6a**,** 6d**, and **6c** demonstrated a remarkable docking scores − 7.30, −7.23, and − 7.12 (k.cal/mol) indicating high binding affinities while **6g**,** 6e**, and **6b** showed moderate to good binding affinities with dock scores − 6.64, −6.80, and − 6.90 k.cal/mol respectively. In brief, the molecular docking results highlight the exceptional binding affinities of synthesized derivatives (Table 6), particularly compounds, **6f**,** 6a**,** 6d**, and **6c** showed the potent binding affinity against DNA gyrase. The specific interactions with key residues provide insights into the molecular mechanisms underlying their potential antibacterial activities.

The molecular docking studies were conducted to elucidate the interactions between the synthesized compounds **(6a-g)** and the bacterial target DNA gyrase, which is crucial for bacterial DNA replication and serves as a significant target for antibacterial agents. The docking results of binding affinity scores and bond lengths provide insights into the binding affinities and molecular interactions of these compounds, correlating with their observed antibacterial and antifungal activities. Notably, compound **6f** (indole-3-ethanamine) exhibited the highest binding affinity of −7.57 k.cal/mol, aligning with its strong antibacterial activity as indicated by both ZOI and MIC values. The presence of multiple hydrogen bond interactions with residues such as GLN 94 and ALA 117 (bond lengths of 2.97 Å and 3.04 Å) suggests effective penetration and stabilization within the active site. Similarly, compound **6g** (thiomorpholine), despite having a slightly lower affinity of −6.64 k.cal/mol, displayed notable antibacterial activity with a high ZOI, indicating that its morpholine group may enhance membrane interactions, supporting its antifungal efficacy as well.

**Table 5:** Molecular Docking energies (k.cal/mol) of the tested (6a-g) ligands.

Compound	rsmd_refine	E_conf	E_refine
**6a**	2.3645	−32.7606	−44.0843
**6b**	1.4026	−67.3454	−31.7621
**6c**	3.4816	−119.5328	−37.8596
**6d**	1.8745	−131.3019	−39.2624
**6e**	1.6810	−185.3885	−32.8882
**6f**	2.1297	−132.1769	−36.6606
**6 g**	1.7305	−45.1289	−29.9268
Streptomycin	1.3066	−105.2598	−38.8258
Ciprofloxacin	1.1013	128.4994	−16.8805
Tetracycline	1.3439	−17.5973	−29.8420
Chloramphenicol	1.1725	100.3267	−27.6884

Rmsd_refine, the root-mean-squared-deviation (RMSD) between the heavy atoms of the predicted pose (after refinement) and those of the crystal structure (before refinement), E_conf, conformer energy in k.cal/mol, E_refine, the score of refinement step of ligand conformer.

Compounds **6a** (N-methylpiperazine), **6d** (4-fluorobenzylamine), and **6c** (piperidone) demonstrated competitive binding affinities to DNA gyrase, with values of −7.30 k.cal/mol, −7.23 k.cal/mol, and − 7.12 k.cal/mol, respectively. The binding affinities of these compounds correlate well with their moderate antibacterial activities, suggesting that their structural features contribute to effective binding, albeit to varying degrees. Specifically, their interactions with multiple residues, including ASP 87 and SER 116 highlight their significant binding potential to the DNA gyrase target, aligning with the observed antibacterial efficacy across these compounds. Conversely, compounds with lower binding affinities, such as **6b** (dimethylamine) and **6e** (cyclopropylamine) demonstrated reduced antibacterial and antifungal activities, which is consistent with their docking scores of −6.90 k.cal/mol and − 6.80 k.cal/mol, respectively. The weaker binding interactions suggest that the less rigid and more flexible structures of these amines hinder optimal binding to target sites, which may contribute to their lower observed activity. The SAR analysis emphasized that the structural characteristics, particularly the presence of rigid and planar substituents like compound **6g** (thiomorpholine) and **6c** (piperidone), are crucial for enhancing both antibacterial and antifungal potencies, as they facilitate stronger interactions with bacterial and fungal targets. Thus, the molecular docking results substantiate the biological activity data, highlighting that strategic modifications in compound structures can optimize their antimicrobial potential. The structure-activity relationship (SAR) derived from the antibacterial and antifungal studies underscores the critical role of amine substituents in influencing biological activity. Compounds such as **6f** (indole-3-ethanamine) and **6c** (piperidone) exhibit rigid and planar structures that enhance binding interactions, resulting in stronger antibacterial and antifungal efficacy. In contrast, more flexible or bulkier groups, as seen in compounds **6b** and **6e**, show reduced potency likely due to steric hindrance and less favorable binding orientations within their target sites. The docking analysis further highlights that specific structural characteristic, including rigidity and electron-donating properties, are pivotal for efficacy; conversely, the presence of electron-withdrawing or bulky groups can negatively impact binding interactions. These insights suggest that strategic modifications to optimize these structural features could significantly enhance both antibacterial and antifungal activities.

**Table 6:**
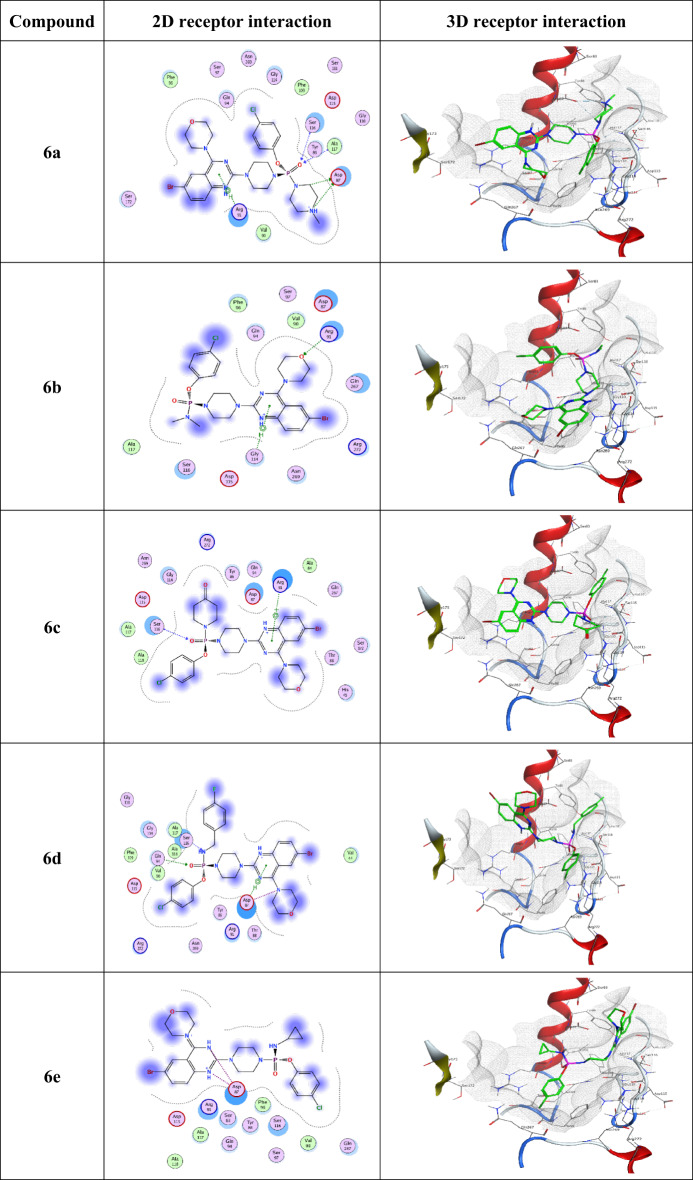

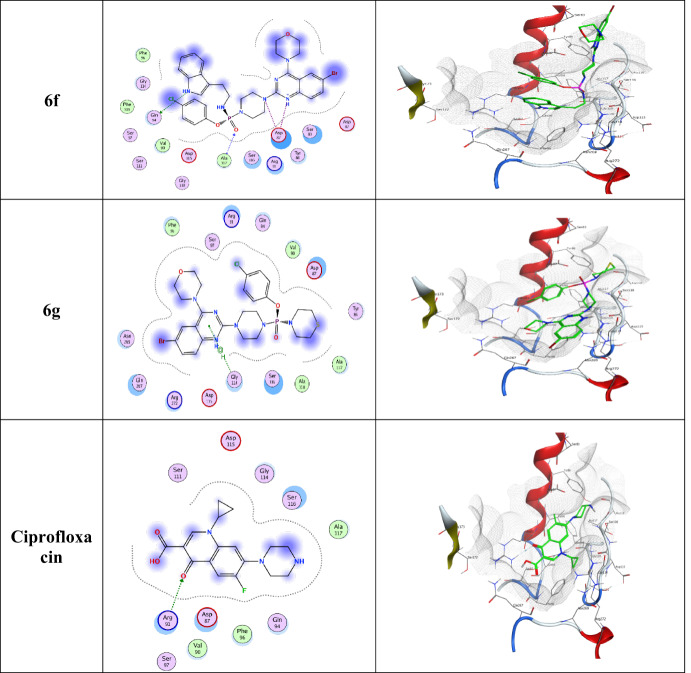
Displays the receptor interaction and position of the most promising possibilities (Compounds 6a-g) within the binding protein pocket about the reference standard of docked ciprofloxacin.

### ADME prediction

The analysis covered several crucial pharmacokinetic parameters, CNS activity score between − 2 and + 2 was used to estimate central nervous system penetration. Most of the test compounds (**6a-g**) showed mild CNS activity (ranging from − 1 to 2), indicating their potential for moderate central distribution (Table 7). In contrast, reference drugs like streptomycin and tetracycline showed a lower CNS activity score of −2. Permeability was measured using two metrics, QPPCaco (Caco-2 cell permeability) and QPPMDCK (MDCK cell permeability). High values in both these parameters indicate efficient cell membrane permeability. Compounds such as **6b**, **6d**, and **6e** demonstrated high permeability with QPPMDCK values of 10,000, far surpassing the reference antibiotics. This suggests that, these compounds are likely to be well-absorbed orally.

**Table 7:** ADME analysis of compounds using QikProp.

Compound	CNS	QPPCaco	QPPMDCK	QPlogBB	QPlogHERG	QPlogKhsa	%HOA	accptHB	donorHB	MW	QPlog Po/w	Rule of Five
**6a**	2	1010.403	3600.163	0.634	−7.014	−0.481	87.777	14.7	0	650.941	3.418	**1**
**6b**	0	4310.69	10,000	0.281	−6.055	−0.423	100	12.7	0	595.862	3.866	**1**
**6c**	−1	1339.799	4415.227	−0.342	−6.43	−0.562	89.984	14.7	0	649.91	3.421	**1**
**6d**	0	4393.656	10,000	0.246	−7.419	0.593	100	12.2	1	675.924	6.082	**2**
**6e**	0	3012.326	10,000	0.024	−6.36	0.215	100	12.2	1	607.873	4.596	**1**
**6f**	−1	2306.102	7979.758	−0.314	−8.03	0.962	100	12.2	2	710.996	6.6	**2**
**6 g**	0	4277.614	10,000	0.38	−6.308	−0.219	100	13.2	0	653.96	4.568	**1**
**Streptomycin**	−2	0.129	0.034	−4.48	−4.991	−1.733	0	25.25	16	581.579	−5.564	**3**
**Ciprofloxacin**	0	12.632	9.192	−0.684	−3.255	0.026	48.297	6	1	331.346	0.28	**0**
**Tetracyclin**	−2	3.859	1.348	−2.096	−5.018	0.023	19.593	8.25	3	444.44	−0.835	**1**
**Chloramphenicol**	**−2**	**80.632**	**201.127**	**−1.4**	**−2.816**	**−0.819**	**67.532**	**6.9**	**3**	**323.132**	**1.104**	**0**

Blood-Brain Barrier Penetration (QPlogBB) showed moderate penetration for most of the tested compounds (values between − 0.342 and 0.634), whereas streptomycin had a notably low score of −4.48, making it less likely to penetrate the CNS. Cardiotoxicity Risk (QPlogHERG), an indicator of hERG channel inhibition, was within acceptable ranges for most new compounds. However, compound **6f** showed a lower QPlogHERG value of −8.03, indicating a potential risk of cardiotoxicity. Solubility (QPlogS) and lipophilicity (QPlogPo/w) were also evaluated. Compound **6f** showed high lipophilicity (6.6), which could impact solubility and bioavailability, while streptomycin displayed poor solubility and lipophilicity (QPlogPo/w = −5.564), possibly limiting its membrane penetration. Human Oral Absorption (%HOA) was excellent for most compounds (around 100%), in stark contrast to streptomycin, which showed 0% oral absorption. Finally, Lipinski’s Rule of Five, a guideline for assessing oral drug-likeness, was applied. All the tested compounds are mostly complied, with **6d** and **6f** each violating two rules, likely due to high molecular weight and hydrogen bond donors. In contrast, ciprofloxacin showed complete compliance (0 violations), highlighting its ideal drug-like properties.

## Experimental section

### Chemistry

Chemicals were obtained from suppliers and used without purification. Reaction progress was monitored using TLC, and compounds were purified through column chromatography using silica gel (60–120 mesh). Melting points were determined using an uncorrected Guna melting point apparatus with open capillaries. FT-IR spectra were recorded using a SHIMADZU-8400 spectrometer. The ^1^H-NMR and ^13^C-NMR, ^31^P-NMR spectra were recorded using JEOL 400 and Bruker Avance 400 spectrometer in CDCl_3_. The Thermo Scientific Flash 2000 Organic Elemental Analyzer with TCD, operated in CHNS/O mode using Eager Experience software, was employed for elemental analysis. Tetramethylsilane is used the internal standard, with data reported in parts per million (*δ*, ppm) and hertz (J = Hz). LCMS spectra was performed using an API 3000 mass spectrometer operating in electrospray positive ionization mode.

#### Synthesis of 6-bromo-2-chloro-4-morpholin-4-yl-quinazoline (2)

Morpholine (1 eq) was added to a stirred solution of 6-bromo-2,4-dichloroquinazoline (**1**, 1 eq) in DCM at 0 °C and stirred at the same temperature for 2 h. The organic layer was washed with satd. NH_4_Cl, brine followed by dried over with anhyd. Na_2_SO_4_, and evaporated by using rota evaporator. The resulting solid was washed with diethyl ether and dried to yield compound **2**.

#### Synthesis of 6-bromo-4-morpholin-4-yl-2-piperazin-1-yl-quinazoline (3)

Compound 2 (1 eq) was dissolved in 1,4-dioxane, followed by the addition of piperazine (1.2 eq) and K_2_CO_3_ (2.5 eq), and the mixture was refluxed to 110 °C for 2 h. After completion, the solvent was removed under vacuum, and the residue was extracted with 10% methanol in chloroform (3 × 15 mL). The combined organic layers were washed with satd. NaHCO_3_ and brine solution followed by dried over with anhyd. Na_2_SO_4_ and solution was removed under reduced pressure. Obtained residue was purified by column chromatography to yield compound **3**.

#### General procedure for the synthesis of title compounds 6(a-g)

Compound **3** (1 eq) was dissolved in THF, then TEA (3 eq) and dichlorophosphinic acid 4-chloro-phenyl ester (1.5 eq) were added successively under N2 atmosphere and stirred at room temperature for 8 h. After completion of the reaction, various substituted 1° & 2° amines (1.1 eq) were added in situ, and the reaction mixture was stirred additional 8 h at same conditions. The completion of the reaction was monitored by TLC, and after dilution with water, the mixture was extracted with 10% methanol in chloroform (3 × 15 mL), followed by drying of the organic layer over anhyd. Na_2_SO_4_ and concentrated under reduced pressure. The obtained crude residue was purified by column chromatography to get the title compounds 6(a-g).

#### 4-Chlorophenyl-4(6-bromo-4-morpholinoquinazolin-2-yl)-piperazin-1-yl-(4-methyl-piperazin-1-yl)-phosphinate (6a)

Yield: 72%, Off White solid, mp: 104–106 °C. FT-IR (KBr, *ν* cm^−1^): 2848(C-H), 1602(C = C), 1543(C = N), 1219(P = O), 1159(P-O-C), 1111(C-O), 1070(C-N), 732(C-Cl), 545(C-Br).^1^H-NMR (400 MHz, CDCl_3_): *δ* 7.65 (s, 1H, CH), 7.47 (d, 1H, *J* = 8 Hz, CH), 7.34–7.28 (m, 1H, CH), 7.26–7.08 (m, 2 H, 2CH), 7.08–6.94 (m, 2 H, 2CH), 4.20–4.07 (m, 4 H, 2CH_2_), 3.85–3.59 (m, 12 H, 6CH_2_), 3.26–3.22 (m, 4 H, 2CH_2_), 3.13–3.01 (m, 4 H, 2CH_2_), 2.15 (S, 3 H, CH_3_). ^13^C-NMR (CDCl_3,_ 100 MHz): *δ* 165.7(C), 161.0(C), 158.3(C), 155.3(C), 135.8(C), 129.7(2 C), 129.3(C-Cl),129.1(C), 129.0(C), 121.5(2 C), 115.7(C), 111.2(C-Br), 66.6(2 C, C-O), 50.4(2 C), 44.8(2 C), 44.7(2 C), 44.4(CH_3_), 44.3(2 C), 44.3(2 C).^31^P-NMR (CDCl_3,_ 125 MHz):*δ*11.53 (P = O). ES-MS (m/z): 650.20 [M(^81^Br) + H]^+^; for C_27_H_34_BrClN_7_O_3_P; Calcd. MW: 649.13. Elemental analysis (%): Found: C 49.67; H 5.12, N 14.93, Calcd: C 49.82; H 5.26, N 15.06.

#### 4-Chlorophenyl-p-4-(6-bromo-4-morpholinoquinazolin-2-yl)-piperazin-1-yl-N, N-dimethyl-phosphonamidate (6b)

Yield: 77%, Off White solid, mp: 164–166 °C. FT-IR (KBr, *ν* cm^−1^):2848(C-H), 1598(C = C), 1541(C = N), 1224(P = O), 1161(P-O-C), 1107 (C-O), 1087(C-N), 736(C-Cl), 642(C-Br).^1^H-NMR (400 MHz, CDCl_3_): *δ* 7.67 (s, 1H, CH), 7.50 (d, 1H, *J* = 8 Hz, CH), 7.28 (d, 2 H, *J* = 8 Hz, 2CH), 7.18–7.13 (m, 3 H, 3CH), 3.87–3.82 (m, 8 H, 4CH_2_), 3.62 (m, 4 H, 2CH_2_), 3.24 (m, 4 H, 2CH_2_), 2.77–2.74 (d, 6 H, J = 12 Hz, 2CH_3_). ^13^C-NMR (CDCl_3,_ 100 MHz):*δ* 165.8(C), 158.5(C), 155.5(C), 150.0(C), 129.7(C-Cl), 128.7(2 C), 127.2(C), 126.3(2 C), 124.2(C), 121.6(C), 121.5(C), 110.7(C-Br), 66.7(2 C, C-O), 50.5(2 C), 44.7(2 C), 44.5(2 C), 44.4(C), 37.0(2 C-NCH_3_). ^31^P-NMR (CDCl_3,_ 125 MHz):*δ*13.51 (P = O). ES-MS (m/z): 597.1 [M(^81^Br) + H]^+^ for C_24_H_29_BrClN_6_O_3_P; Calcd. MW: 595.86. Elemental analysis (%): Found: C 48.29; H 4.85, N 13.98; Calcd: C 48.38; H 4.91, N 14.10.

#### 4-Chlorophenyl-4-(6-bromo-4-morpholinoquinazolin-2-yl)-piperazin-1-yl-(4-oxo-piperidin-1-yl)-phosphinate (6c)

Yield: 70%, Off White solid, mp: 93–95 °C. FT-IR (KBr, *ν* cm^−1^):2850(C-H), 1716(C = O), 1600(C = C), 1539(C = N), 1207(P = O), 1161(P-O-C), 1112(C-O), 1089(C-N), 729(C-Cl), 684(C-Br).^1^H-NMR (400 MHz, CDCl_3_): *δ* 7.68 (s, 1H, CH), 7.5 (d, 1H, *J* = 12 Hz, CH), 7.31–7.21 (m, 4 H, 4CH), 7.17–7.50 (m, 1H, CH), 3.95–3.80 (m, 8 H, 4CH_2_), 3.64–3.61 (m, 4 H, 2CH_2_), 3.55–3.50 (m, 4 H, 2CH_2_), 3.39–3.28 (m, 4 H, 2CH_2_), 2.46–2.44 (m, 4 H, 2CH_2_). ^13^C-NMR (CDCl_3,_ 100 MHz):*δ* 184.2(C = O), 165.7(C), 158.3(C), 149.7(C), 149.6(C), 130.1(C), 129.9(2 C), 128.7(C-Cl), 127.3(C), 126.3(C), 124.4(2 C), 121.4(C), 110.7(C-Br), 66.7(2 C, C-O), 50.4(2 C), 45.2(C), 45.1(C), 44.9(2 C), 44.4(2 C), 42.4(C), 42.3(C). ^31^P-NMR (CDCl_3,_ 125 MHz): *δ* 10.56 (P = O). ES-MS (m/z): 651.1 [M (^81^Br) + H]^+^ for C_27_H_31_BrClN_6_O_4_P; Calcd. MW: 649.90. Elemental analysis (%): Found: C 49.81; H 4.76, N 12.84; Calcd: C 49.90; H 4.81, N 12.93.

#### 4-Chlorophenyl-p-4-(6-bromo-4-morpholinoquinolin-2-yl)-piperazin-1-yl-N-(4-fluorobenzyl)-phosphonamidate (6d)

Yield: 75%, Dark brown solid, mp: 114–116 °C. FT-IR (KBr, *ν* cm^−1^):3194(N-H), 2856(C-H), 1600(C = C), 1548(C = N), 1209(P = O), 1159(P-O-C), 1112(C-O), 1083(C-N), 763(C-Cl), 607(C-Br). ^1^H-NMR (400 MHz, CDCl_3_): *δ*8.21 (s, 1H, CH), 7.71 (d, 2 H, *J* = 8 Hz, 2CH), 7.38–7.34 (m, 4 H, 4CH), 7.19–7.04 (m, 4 H, 4CH), 4.21 (d, 2 H, CH_2_), 3.81–3.74 (m, 8 H, 4CH_2_), 3.21–3.16 (m, 4 H, 2CH_2_), 2.83–2.76 (m, 4 H, 2CH_2_). ^13^C-NMR (CDCl_3,_ 100 MHz):*δ* 165.6(C), 158.4(C), 155.4(C-F), 149.8(C), 131.3(2 C), 130.1(C-Cl), 129.7(2 C), 128.6(2 C), 126.2(3 C), 126.9(C), 121.6(C), 121.5(C), 115.3 (2 C), 110.6(C-Br), 66.6(2 C, C-O), 55.1(2 C), 50.4(2 C), 46.3(2 C), 44.7(C). ^31^P-NMR (CDCl_3,_ 125 MHz):*δ*11.89 (P = O). ES-MS (m/z): 677.1 [M(^81^Br) + H]^+^ for C_29_H_30_BrClFN_6_O_3_P; Calcd. MW: 675.92. Elemental analysis (%): Found: C 51.39; H 4.41, N 12.27; Calcd: C 51.53; H 4.47, N 12.43.

#### 4-Chlorophenyl-p-4-(6-bromo-4-morpholinoquinazolin-2-yl)-piperazin-1-yl-N-cyclopropylphosphonamidate (6e)

Yield: 72%, Off white solid, mp: 89–91 °C. FT-IR (KBr, *ν* cm^−1^):3197(N-H), 2848(C-H), 1600(C = C), 1539(C = N), 1209(P = O), 1161(P-O-C), 1111(C-O), 1089(C-N), 785(C-Cl), 684(C-Br).^1^H-NMR (400 MHz, CDCl_3_): *δ* 7.68 (s, 1H, CH), 7.51 (d, 1H, *J* = 12 Hz, CH), 7.28–7.26 (m, 2 H, 2CH), 7.19–7.13 (m, 3 H, 3CH), 3.88–3.79 (m, 8 H, 4CH_2_), 3.64–3.61 (m, 4 H, 2CH_2_), 3.30–3.26 (m, 4 H, 2CH_2_), 3.13 (d, 1H, N-H), 2.47–2.45 (m, 1H, CH), 0.78 − 0.42 (m, 4 H, 2CH_2_). ^13^C-NMR (CDCl_3,_ 100 MHz):*δ* 165.7(C), 158.4(C), 155.4(C), 149.8(C), 149.7(C), 129.7(2 C), 128.7(C-Cl), 127.2(C), 126.3(C), 124.2(2 C), 121.6(C), 110.6(C-Br), 66.7(2 C, C-O), 50.4(2 C), 44.7(2 C), 44.4(2 C), 22.7(C), 7.3(2 C). ^31^P-NMR (CDCl_3,_ 125 MHz): *δ* 10.69 (P = O).ES-MS (m/z): 609.0 [M(^81^Br) + H]^+^ for C_25_H_29_BrClN_6_O_3_P; Calcd. MW: 607.87. Elemental analysis (%): Found: C 49.27; H 4.76, N 13.69; Calcd: 49.40; H 4.81, N 13.83.

#### 4-Chlorophenyl-N−2-(1 h-indol-3-yl)-ethyl-p-(4-(6-bromo-4-morpholinoquinazolin-2-yl)-piperazin-1-yl)-phosphonamidate (6f)

Yield: 68%, Off white solid, mp: 99–101 °C. FT-IR (KBr, *ν* cm^−1^):3184(N-H), 2852(C-H), 1600(C = C), 1539(C = N), 1217(P = O), 1160 (P-O-C), 1112(C-O), 1089(C-N), 738(C-Cl), 684(C-Br). ^1^H-NMR (400 MHz, CDCl_3_): *δ* 8.32 (d, 1H, J = 8 Hz, CH), 7.68 (s, 1H, CH), 7.58–7.48 (m, 2 H, 2CH), 7.36–7.31 (m, 1H, CH), 7.27–7.08 (m, 5 H, 5CH), 7.03–7.01 (m, 1H, CH), 6.89–6.83 (m, 1H, CH), 3.87–3.61 (m, 4 H, 2CH_2_), 3.42–3.18 (m, 4 H, 2CH_2_), 3.01–2.97 (m, 4 H, 2CH_2_), 2.86–2.83 (m, 4 H, 2CH_2_), 2.78–2.72 (m, 2 H, CH_2_), 2.67–2.61 (m, 2 H, CH_2_). ^13^C-NMR (CDCl_3,_ 100 MHz):*δ* 165.7(C), 158.4(C), 149.8(C), 136.6(C), 136.5(C), 129.7(C), 129.6(C), 128.6(C-Cl), 127.3(C), 127.3(C), 127.2(C), 126.3(C), 124.2(C), 122.6(C), 121.8(2 C), 119.6(C), 118.7(2 C), 112.4(C), 111.5(C), 110.6(C-Br), 66.7(2 C, C-O), 50.4(2 C), 44.8(C), 44.5(C), 44.4(C), 41.4(C), 41.3(C), 27.6(C). ^31^P-NMR (CDCl_3,_ 125 MHz):*δ*12.0 (P = O). ES-MS (m/z): 712.1 [M(^81^Br) + H]^+^ for C_25_H_29_BrClN_6_O_3_P; Calcd. MW: 711. Elemental analysis (%): Found: C 53.92; H 4.75, N 13.58; Calcd: C 54.06; H 4.82, N 13.79.

#### 4-Chlorophenyl-4-(6-bromo-4-morpholinoquinazolin-2-yl)-piperazin-1-yl-(thiomorpholino)-phosphinate (6g)

Yield: 76%, Off-white solid, mp: 120–122 °C. FT-IR (KBr, *ν* cm^−1^): 2848(C-H), 1600(C = C), 1539(C = N), 1215(P = O), 1163(P-O-C), 1112(C-O), 1085(C-N), 767(C-Cl), 684(C-Br). ^1^H-NMR (400 MHz, CDCl_3_): *δ* 7.68 (s, 1H, CH), 7.50 (d, 1H, J = 12 Hz, CH), 7.30–7.27 (m, 2 H, 2CH), 7.20–7.14 (m, 3 H, 3CH), 3.87–3.83 (m, 8 H, 4CH_2_), 3.63–3.61 (m, 4 H, 2CH_2_), 3.50–3.43 (m, 4 H, 2CH_2_), 3.41–3.23 (m, 4 H, 2CH_2_), 2.58–2.54 (m, 4 H, 2CH_2_). ^13^C-NMR (CDCl_3,_ 100 MHz):*δ* 165.7(C), 158.4(C), 155.4(C), 149.8(C), 149.7(C), 129.8(2 C), 128.7(C-Cl), 127.2(C), 126.3(C), 124.3(2 C), 121.5(C), 110.7(C-Br), 66.7(2 C, C-O), 50.4(2 C), 46.9(2 C), 44.8(2 C), 44.4(2 C), 27.5(2 C-S). ^31^P-NMR (CDCl_3,_ 125 MHz):*δ*10.72 (P = O). ES-MS (m/z): ES-MS (m/z): 655.1 [M(^81^Br) + H]^+^, for C_26_H_31_BrClN_6_O_3_PS; Calcd. MW: 653.96. Elemental analysis (%): Found: C 47.58; H 4.66, N 12.53, S 4.73; Calcd: C 47.75; H 4.78, N 12.85, S 4.90.

### Biological activity

#### Antibacterial activity

Each of the tested compounds (200 *µg*) was dissolved in DMSO (1 mL). Centrifuged pellets of bacteria from 24 h old culture containing approximately 104–106 colony forming units (CFU) per mL were spread on the surface of nutrient agar plates. Nutrient agar medium was prepared by suspending nutrient agar (28 g) in distilled water (1 L), autoclaved, and cooled to 45 °C. Then the agar was seeded with 15 mL of prepared inocula of the bacteria to obtain concentration 106 CFU/mL. Petri dishes were prepared by pouring 10 mL of seeded nutrient agar. Wells were created in the medium with the help of a sterile metallic borer, and a test solution was added into each of them. The experimental plates were incubated for 24 h at 37 °C, and the diameter of the inhibition zone around each well was measured^35^.

#### **Determination of minimum inhibitory concentration (MIC**)

Antimicrobial activity of test compounds **(6a-g)** was tested against the test cultures by the macrobroth dilution method using 13 sterile test tubes. 1 ml of Mueller Hinton Broth was added to each tube. 1 ml of the extract was added to tubes 1 and 2. Tubes 2 to 10 were serially diluted by removing 1 ml from tube 2 then subsequently transferring 1 ml to the succeeding tubes until tube10. 1 ml from tube 10 was discarded. 1 ml bacterial suspension was added to all 13 tubes except for tube 12 which served as the negative control. 1 ml of 30 µg/ml of Amoxyclav standard was added to tube 13. The tubes were incubated for 24 h at 37 ^o^C. The optical density values at 600 nm were determined using a UV/Vis Spectrophotometer. The MIC was determined as the lowest concentration of the test samples **6a-g** that visually inhibits the microbial growth in comparison with the positive control (Amoxyclav) as reflected by a decrease in absorbance reading as compared to the initial absorbance (Prepare the standardized inoculums at OD600 = 0.063 in 0.85% w/v sterile saline)^35^.

#### Antifungal activity

Test compounds **6a**, **6c**, **6e**, **6f** and **6 g** were dissolved in DMSO before mixing with potato dextrose agar (PDA). The final concentration of the compounds in the medium was fixed at 200 *µ*g/mL. Two kinds of fungi were incubated in PDA at 25 ± 1 °C for 5 days to get new mycelium for antifungal assay, and then a mycelium disk of approximately 0.45 cm diameter, cut from the culture medium, was picked up with a sterilized inoculation needle and inoculated in the center of the PDA plate. The inoculated plates were incubated at 25 ± 1 °C for 5 days. DMSO was added as the negative control to determine possible inhibitory activity of the solvent, while Fluconazole was used as the positive control. For each treatment, the experiment was repeated three times and the mean value of the diameter of the inhibition zones was calculated^36,37^.

#### Docking studies

Docking studies using the Molecular Operating Environment (MOE) software package 2015. The docking study used DNA gyrase A PDB D: 1AB4 to examine the mode of action of the small molecule compounds as antimicrobial agents. The crystal structure of the 59KDA fragment of gyrase from *Escherichia coli* was obtained from Protein Data Bank (https://www.rcsb.org/*).* The compounds were drawn using ChemDraw, brought into MOE, and then adjusted in 3D for protons and energy until the gradient reached 0.01, and saved as an MDB file for docking calculations. The protein structure is imported into MOE and the structure preparation wizard of MOE was used to correct all the issues in protein structures. The hydrogen atoms were added to structures in their standard geometry, and all solvent molecules were removed from the structures and then subjected to energy minimization. The final optimized structures were saved in the working directory. The program specifications were set up as follows: dummy atoms act as the docking site, triangle matcher is the placement methodology, London dG is the scoring methodology, rigid receptor represents the refinement methodology, and GBVI/WSA dG is the scoring methodology for selection of the best poses.The scoring methods were adjusted to default values. The MDB file of the three ligands was loaded and general dock calculations were run automatically. After completion of docking processes, the obtained poses were studied^38^.

#### ADME study

The ADME (Absorption, Distribution, Metabolism, and Excretion) prediction process involved a comprehensive assessment of the selected ligands’ pharmacokinetic properties.Lipinski’s Rule of Fiveof the compounds were evaluated using QikProp^39^. The ADME properties of the target derivatives **6a–g**were compared with standard antibiotics such as streptomycin, ciprofloxacin, tetracycline, and chloramphenicol were evaluated using the QikProp module in Schrödinger. This analysis helps to determine the drug-likeness and pharmacokinetic feasibility of the compounds for potential therapeutic use. Various parameters, including CNS activity, human oral absorption (HOA), permeability (QPPCaco, QPPMDCK), lipophilicity (QPlogPo/w), solubility (QPlogS), blood-brain barrier penetration (QPlogBB), and potential cardio toxicity (QPlogHERG), were analyzed to assess the pharmacokinetic suitability of the compounds.

## Conclusion

A series of novel quinazoline-phosphorodiamidate-linked piperazine hybrids **(6a-g)** are synthesized with good yields and screened for their antimicrobial activity against the growth of selected microorganisms. In the biological assay, compounds **6b**,** 6d**, and **6e** showed good antibacterial and the compounds **6a**,** 6c**,** 6f**, and **6 g** showed potent antibacterial activity against tested bacterial strains. Compounds **6a** and **6e** showed good antifungal activity and compounds **6c**,** 6f**, and **6 g** showed potent antifungal activity against tested fungal strains. The potency of compounds**6f**,** 6a**,** 6c**, and **6 g** was further substantiated by molecular docking studies, which demonstrated robust binding affinity to DNA gyrase, as evidenced by a superior docking score and critical interactions with key active site residues, underscoring its potential as a potent antimicrobial activity against tested pathogens. Further, in silico ADMET prediction studies showed that most of the potent analogues are CNS active with a minor deviation from Lipinski’s rules.To further explore the therapeutic promise of these quinazoline-piperazine phosphonodiamidite derivatives, future work will focus on in vivo efficacy studies to provide deeper insights into their biological activities, safety profile, and therapeutic relevance in a physiological context.

## Electronic supplementary material

Below is the link to the electronic supplementary material.

Supplementary Material 1

## Data Availability

All data generated or analyzed during this study are included in the published article, and supplementary files are available upon request. Any raw data or additional files in a different format can be obtained from the corresponding author upon reasonable request.
