# Supplementary material for: Design, synthesis, and antimicrobial evaluation of novel quinazoline piperazine phosphorodiamidate hybrids as potent DNA gyrase inhibitors
Source: Sci Rep. 2025 Sep 30;15:33964. doi: 10.1038/s41598-025-11516-7 (PMC12484721; doi:10.1038/s41598-025-11516-7)
Supplement: Supplementary file 1 — Supplementary Material 1 [file 41598_2025_11516_MOESM1_ESM.docx]

**DESIGN, SYNTHESIS, AND ANTIMICROBIAL EVALUATION OF NOVEL QUINAZOLINE PIPERAZINE PHOSPHORODIAMIDATE HYBRIDS AS POTENT DNA GYRASE INHIBITORS**

Suresh Babu Donka ^a^, Sajitha Kethineni ^a^, Bal Yesu Valaparla ^a&b^, Anusha Bhimreddy ^c^, Manjunadh D Meti ^d^, Uttam A More ^e^, Venkata Subbaiah Kotakadi ^f^, Murali Vatturu ^a^, Srinivasulu Doddaga *^a^

^a^ Department of Chemistry, Sri Venkateswara University, Tirupati-517502, Andhra Pradesh, India

^b^ Department of Chemistry, SGK Government Degree College, Vinukonda, Palanadu-522647, Andhra Pradesh, India

^c^ Department of Chemistry, Silver Jubilee Government College, Cluster University, Kurnool-518502, Andhra Pradesh, India

^d^ Department of Plant Sciences, School of Life Sciences, University of Hyderabad, Gachibowli-500046, Telangana, India

^e^ Department of Pharmaceutical Chemistry, Shree Dhanvantary Pharmacy College, Kim, Suram-394110, India

^f^ Department of Biochemistry, Sri Venkateswara University, Tirupati-517502, Andhra Pradesh, India

^*^Corresponding Author: Srinivasulu Doddaga, Department of Chemistry, Sri Venkateswara University, Tirupati-517502, Andhra Pradesh, India.

Tel: +91-949316701; E-mail: [doddaga_s@yahoo.com](mailto:doddaga_s@yahoo.com)

**Supporting Data for the Compounds (6a-g) – FT-IR, ^1^H-NMR, ^13^C-NMR, ^31^P-NMR, LC-MS and CHNO.**


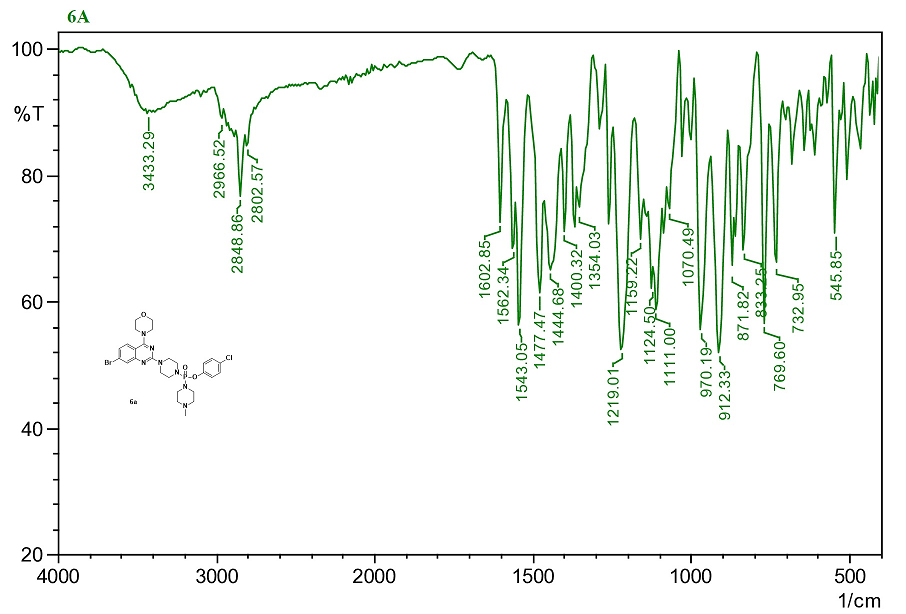


**Figure S1.** FT-IR Spectrum of Compound **6a**


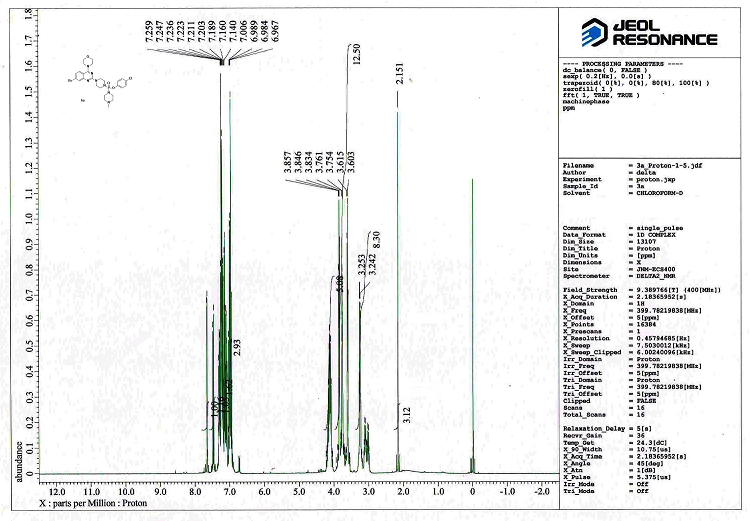


**Figure S2.** ^1^H-NMR Spectrum of Compound **6a**


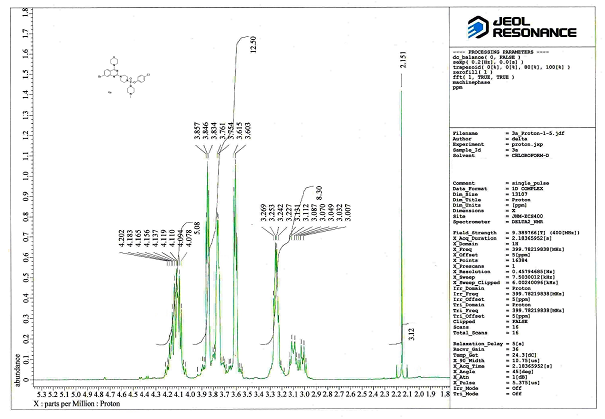


**Figure S3.** ^1^H-NMR Spectrum of Compound **6a (Closure view of aliphatic region)**


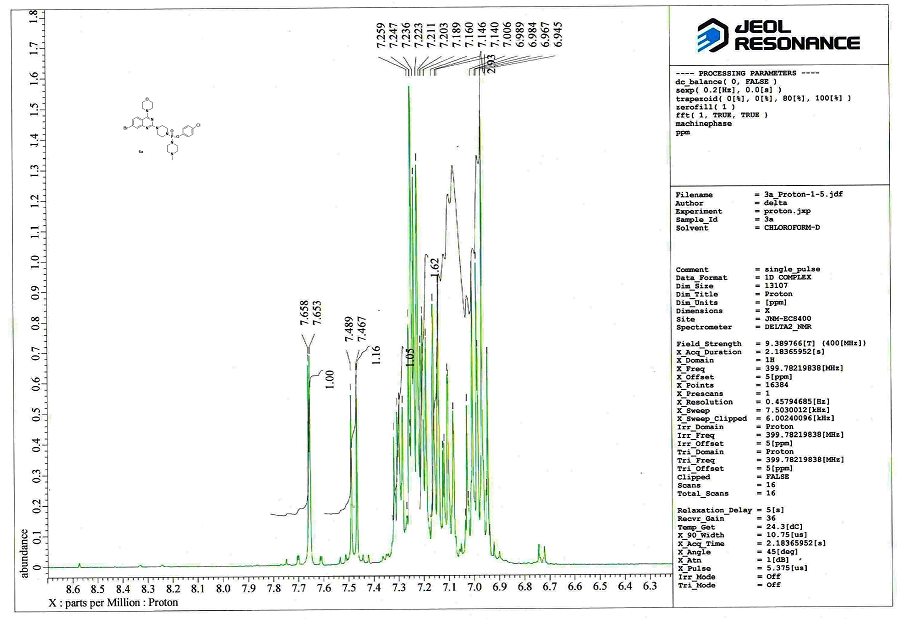


**Figure S4.** ^1^H-NMR Spectrum of Compound **6a (Closure view of aromatic region)**


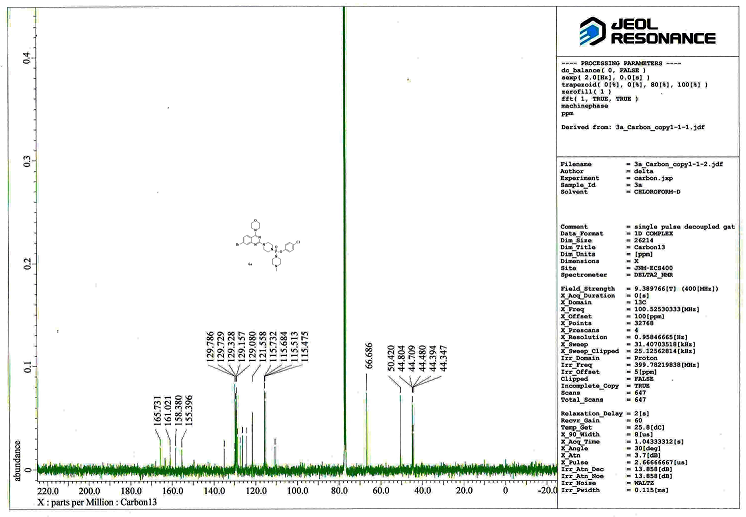


**Figure S5.** ^13^C-NMR Spectrum of Compound **6a**


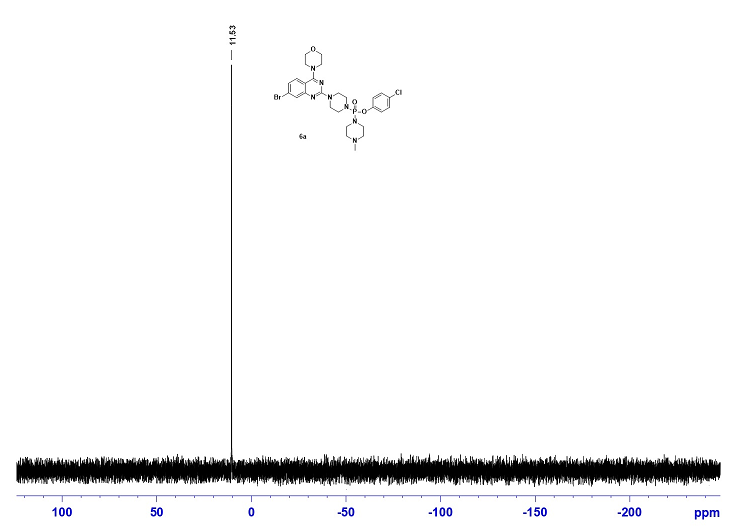


**Figure S6.** ^31^P-NMR Spectrum of Compound **6a**


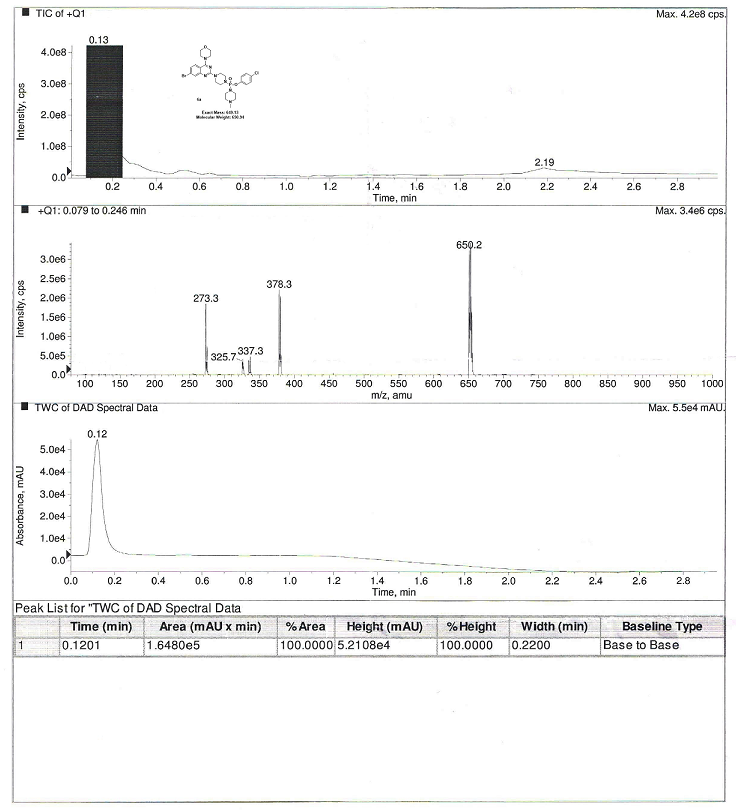


**Figure S7.** LC-MS Spectrum of Compound **6a**


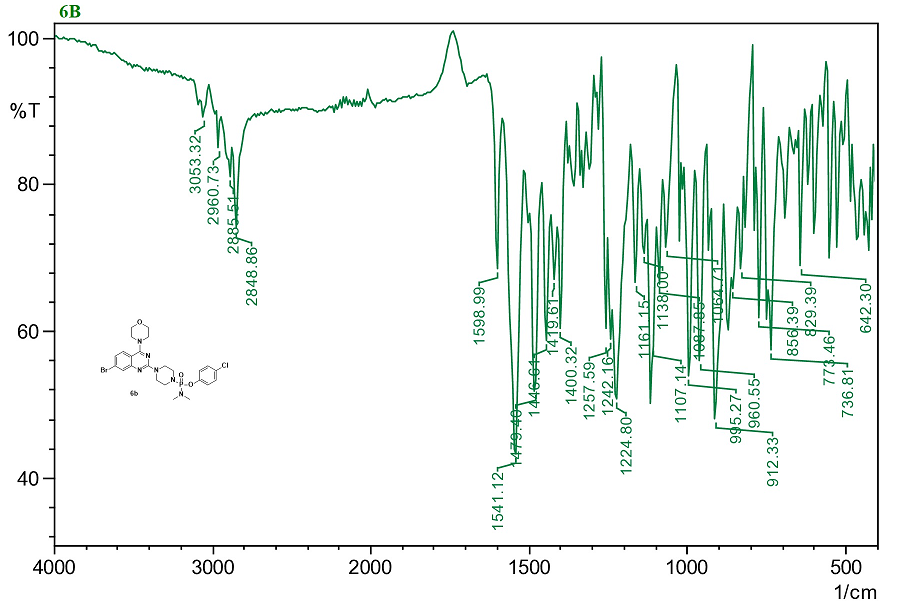


**Figure S8.** FT**-**IR Spectrum of Compound **6b**


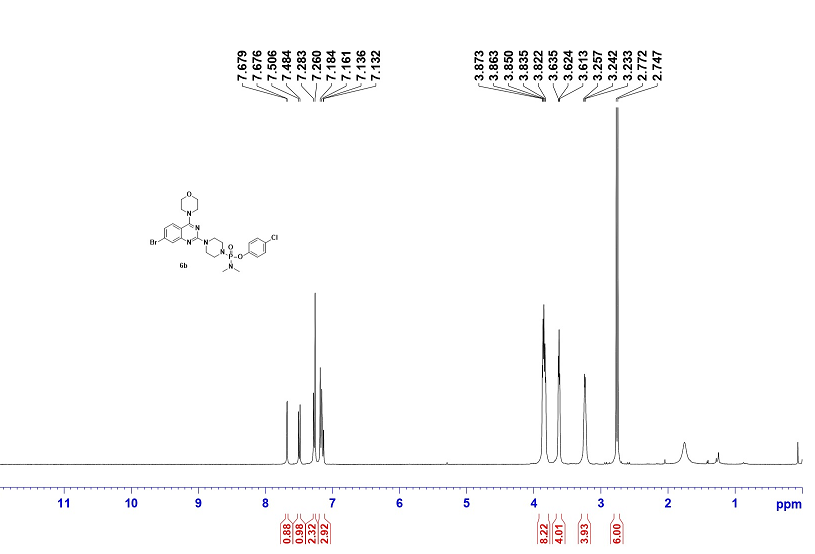


**Figure S9.** ^1^H-NMR Spectrum of Compound **6b**


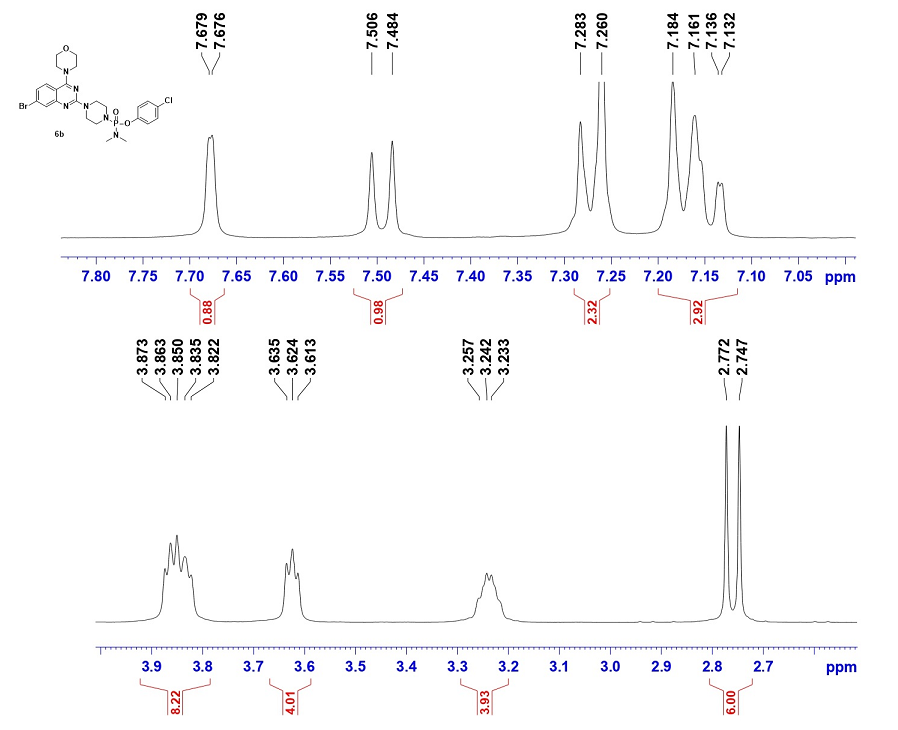


**Figure S10.** ^1^H-NMR Spectrum of Compound **6b (Closure view)**


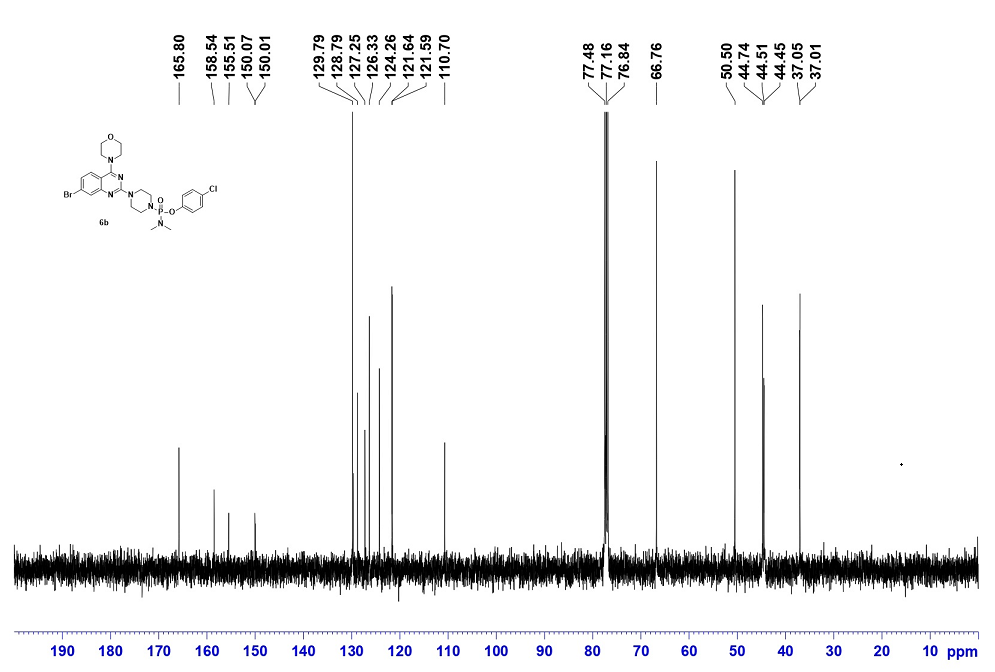


**Figure S11.** ^13^C-NMR Spectrum of Compound **6b**


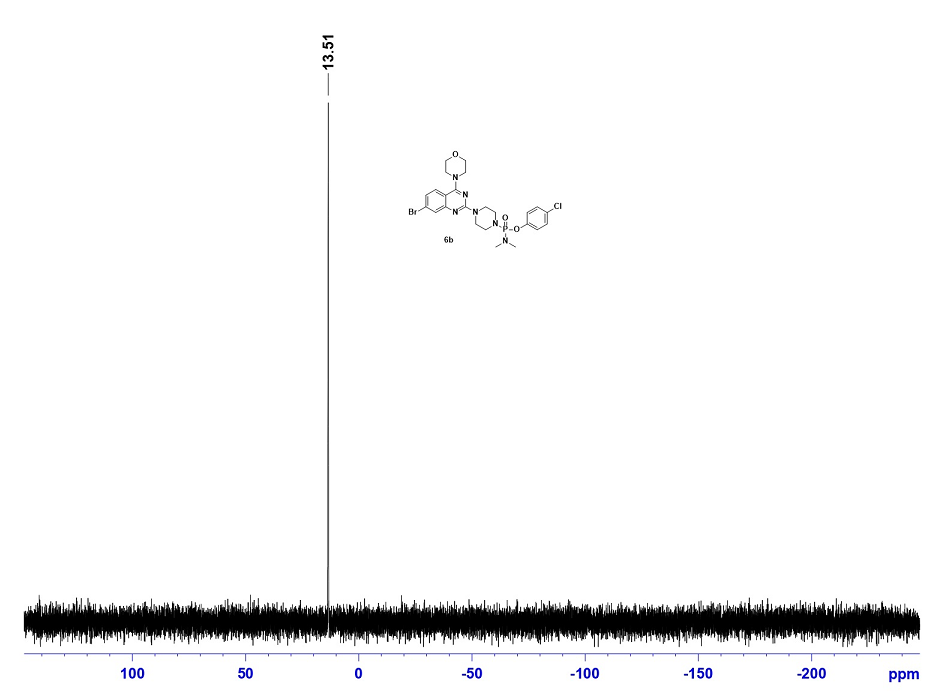


**Figure S12.** ^31^P-NMR Spectrum of Compound **6b**


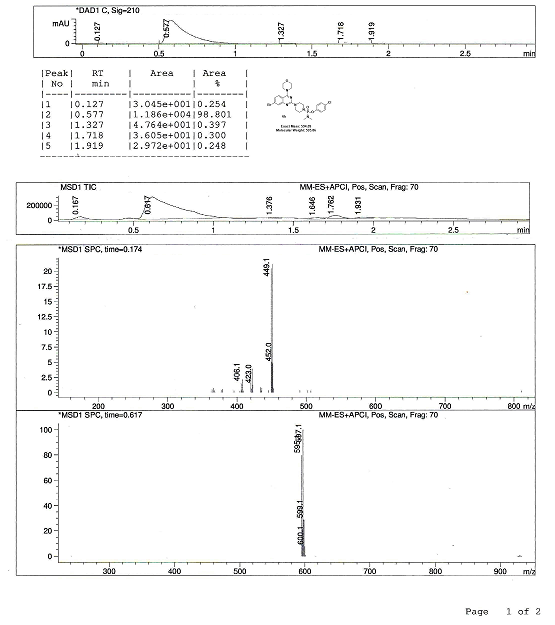

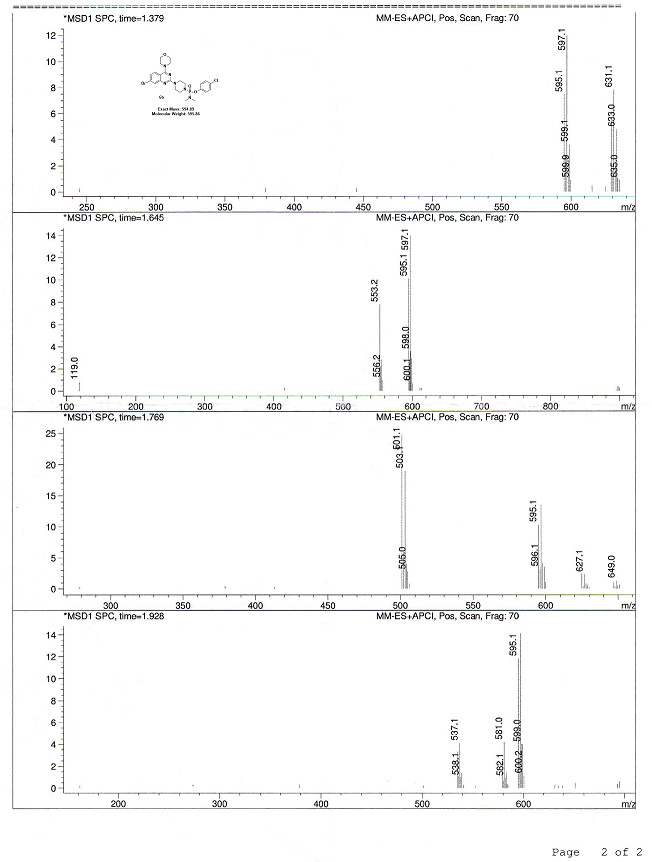


**Figure S13.** LC-MS Spectrum of Compound **6b**


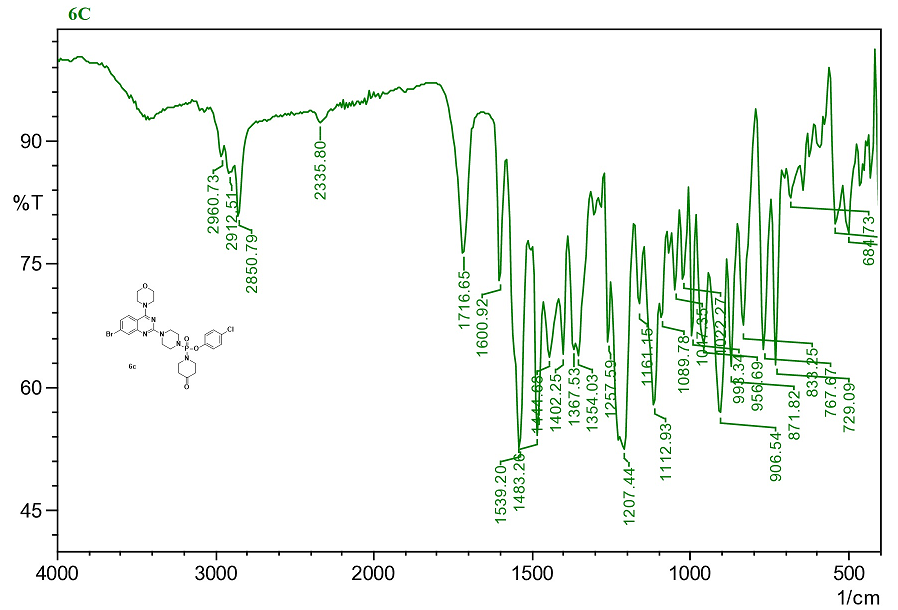


**Figure S14.** FT**-**IR Spectrum of Compound **6c**


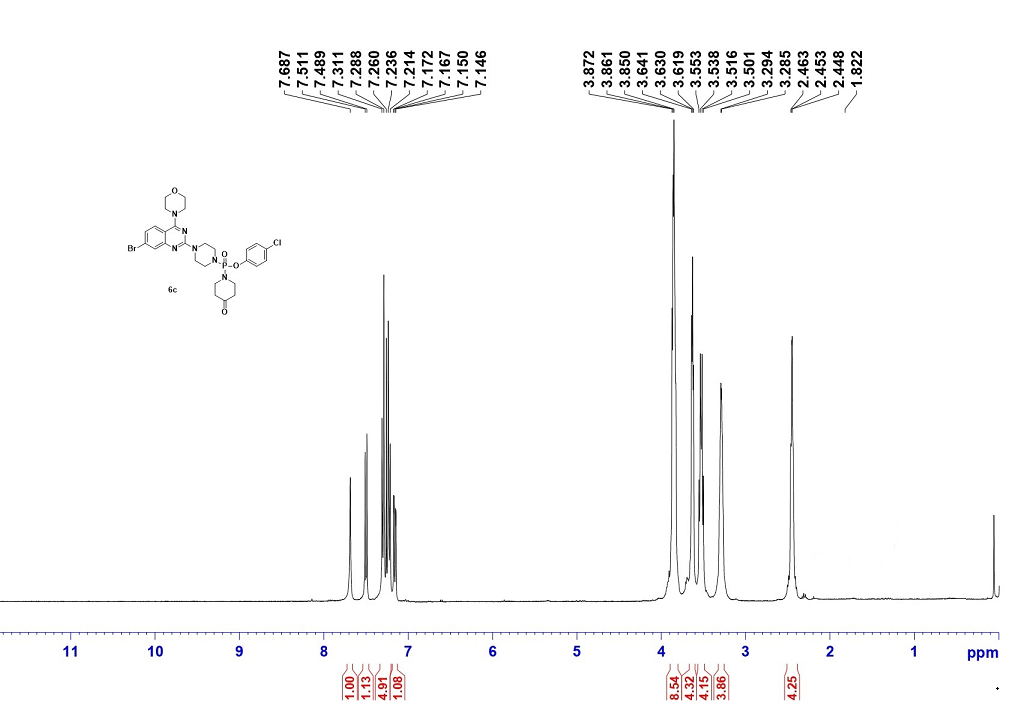


**Figure S15.** ^1^H-NMR Spectrum of Compound **6c**


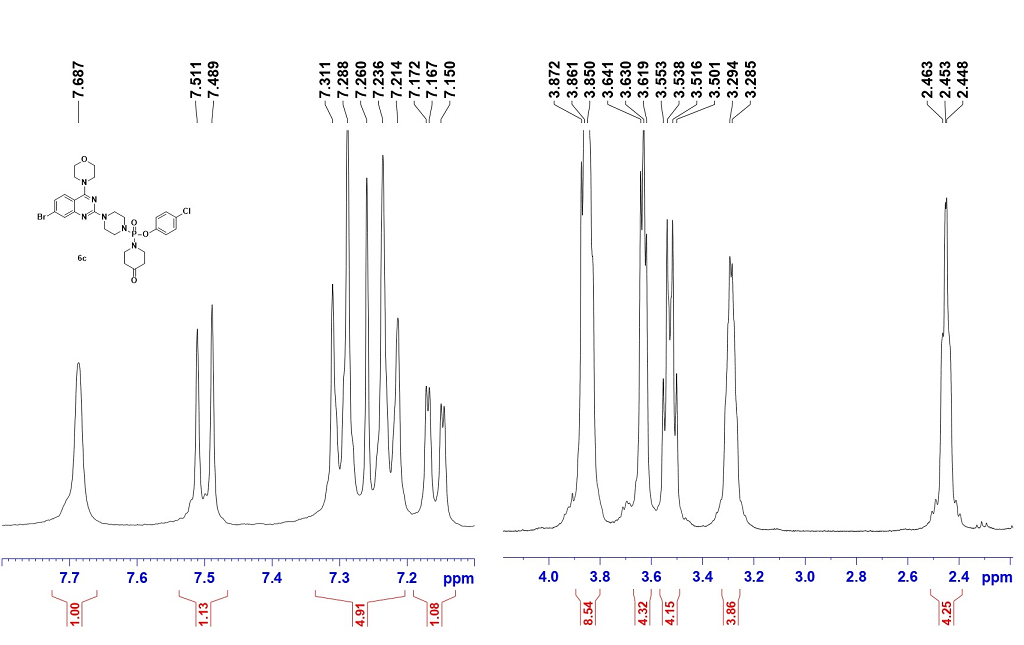


**Figure S16.** ^1^H-NMR Spectrum of Compound **6c**


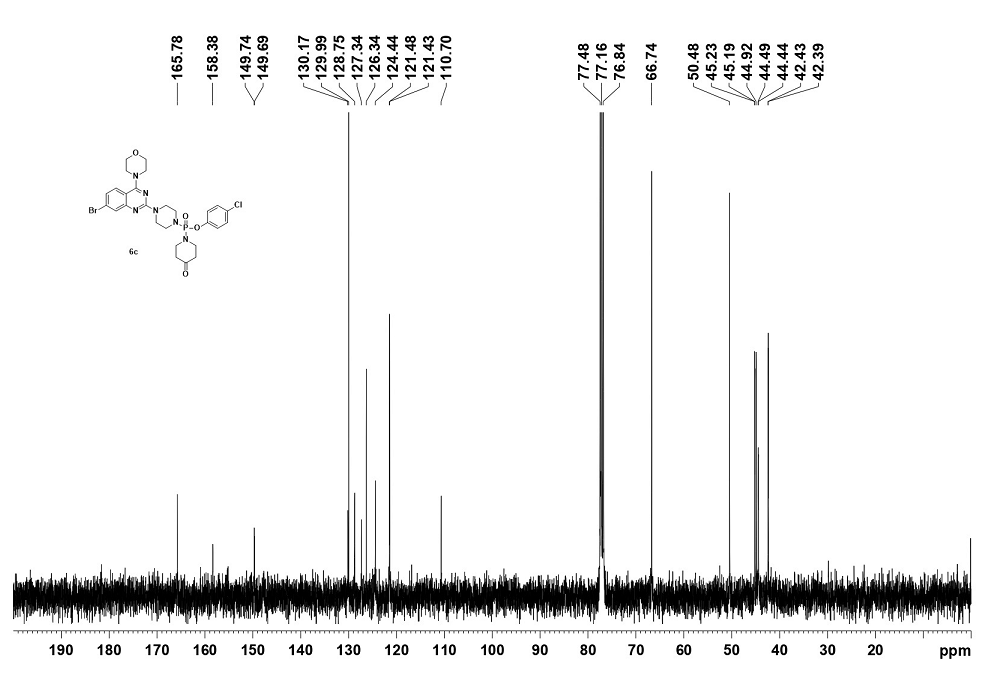


**Figure S17.** ^13^C-NMR Spectrum of Compound **6c**


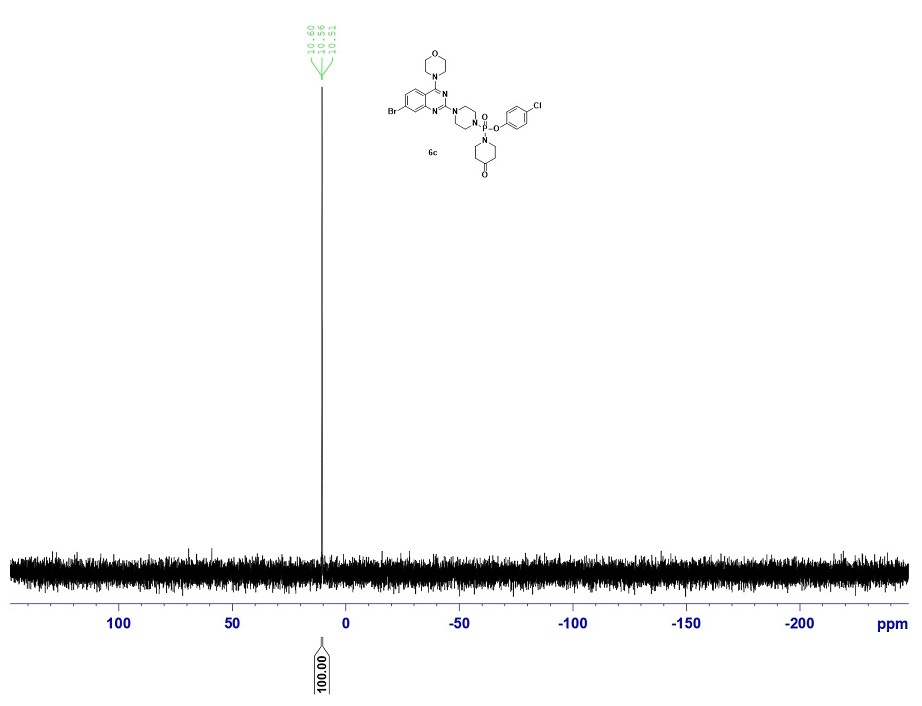


**Figure S18.** ^31^P-NMR Spectrum of Compound **6c**


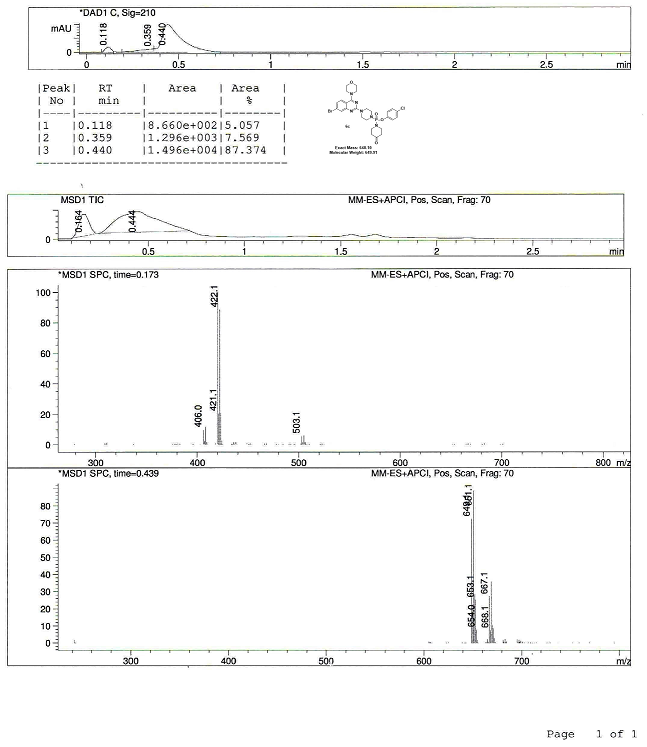


**Figure S19.** LC-MS Spectrum of Compound **6c**


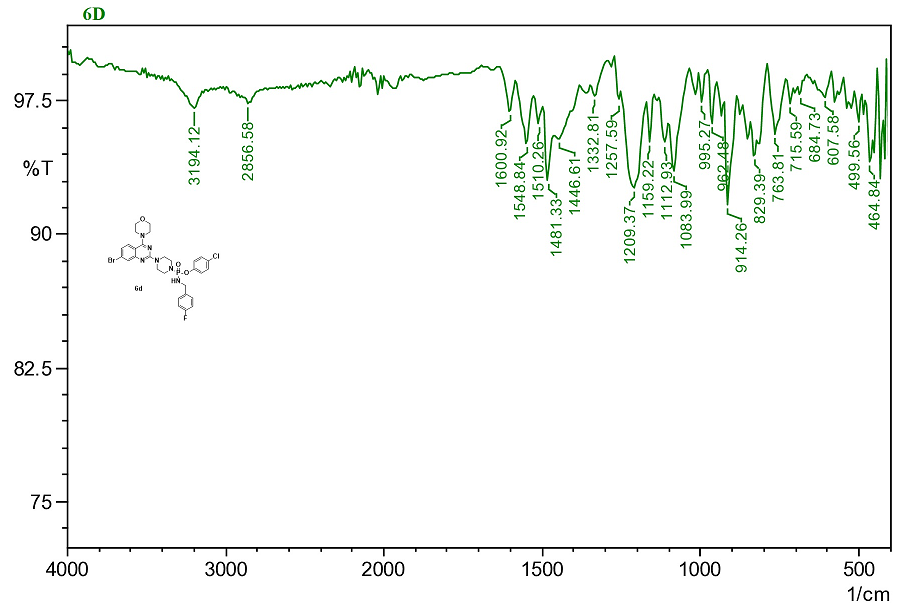


**Figure S20.** FT**-**IR Spectrum of Compound **6d**


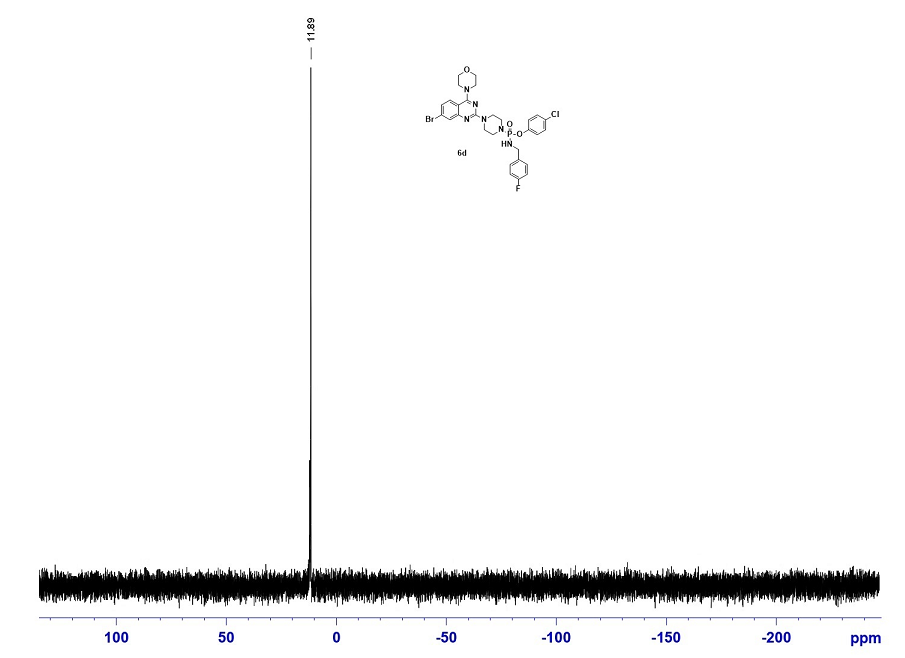


**Figure S21.** ^31^P-NMR Spectrum of Compound **6d**


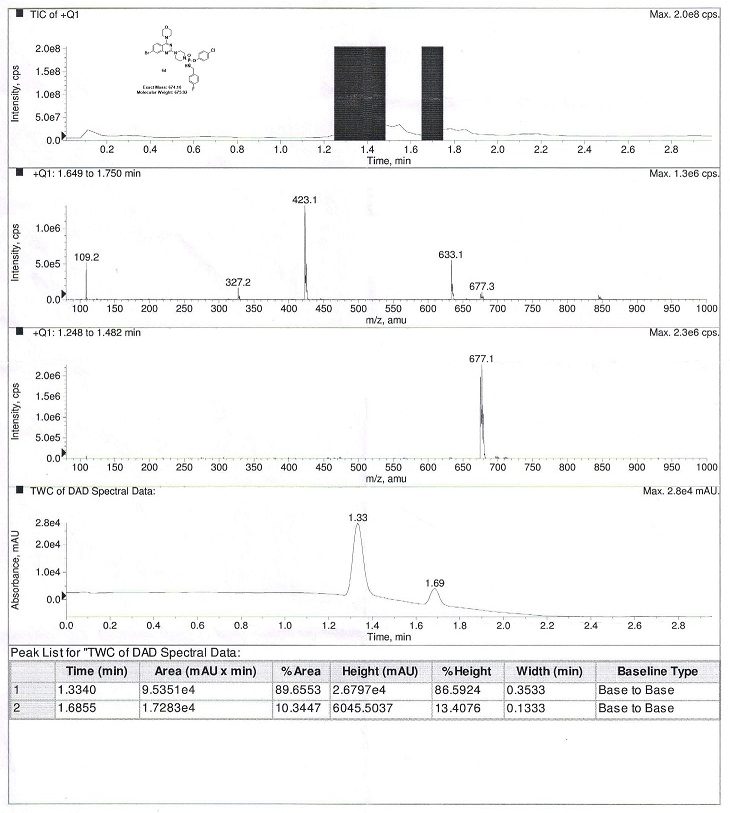


**Figure S22.** LC-MS Spectrum of Compound **6d**


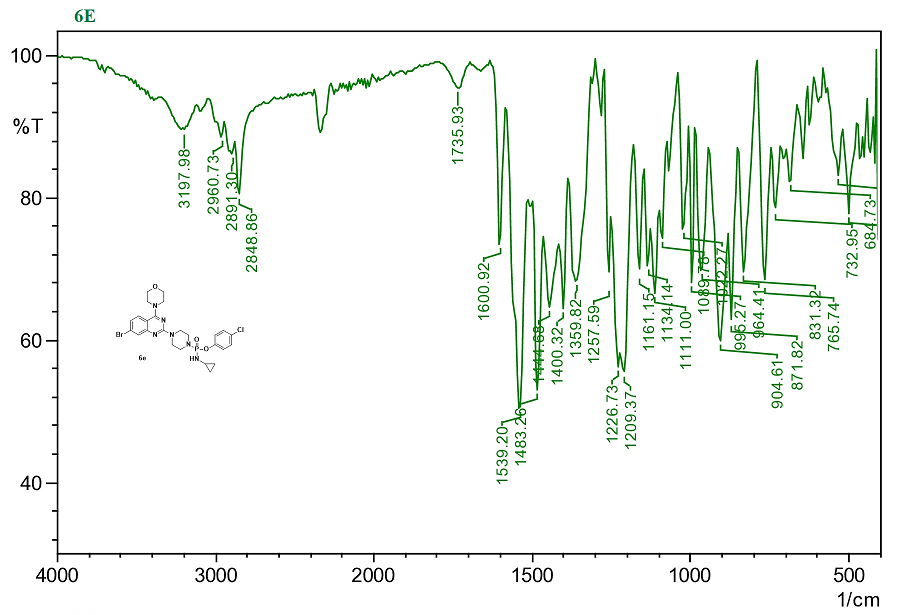


**Figure S23.** FT**-**IR Spectrum of Compound **6e**


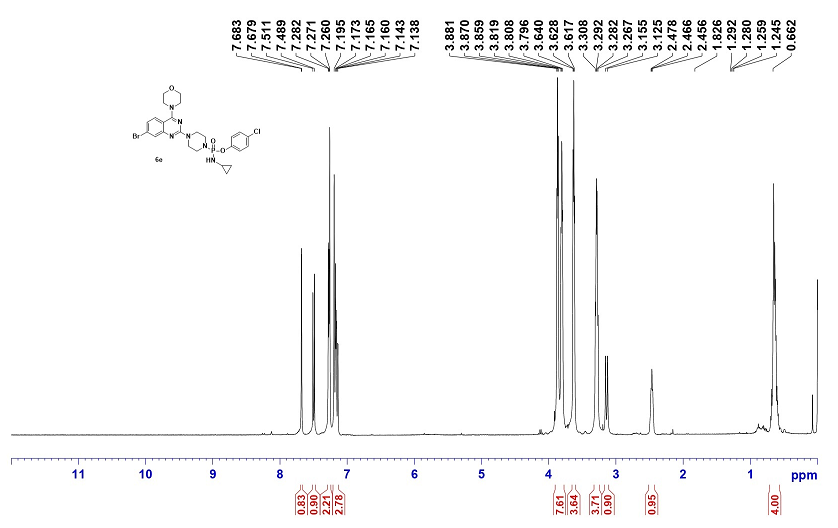


**Figure S24.** ^1^H-NMR Spectrum of Compound **6e**


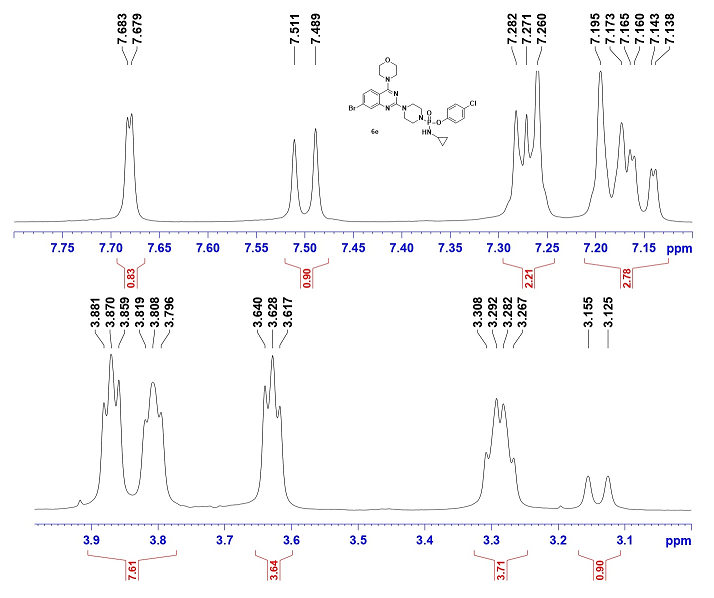


**Figure S25.** ^1^H-NMR Spectrum of Compound **6e**


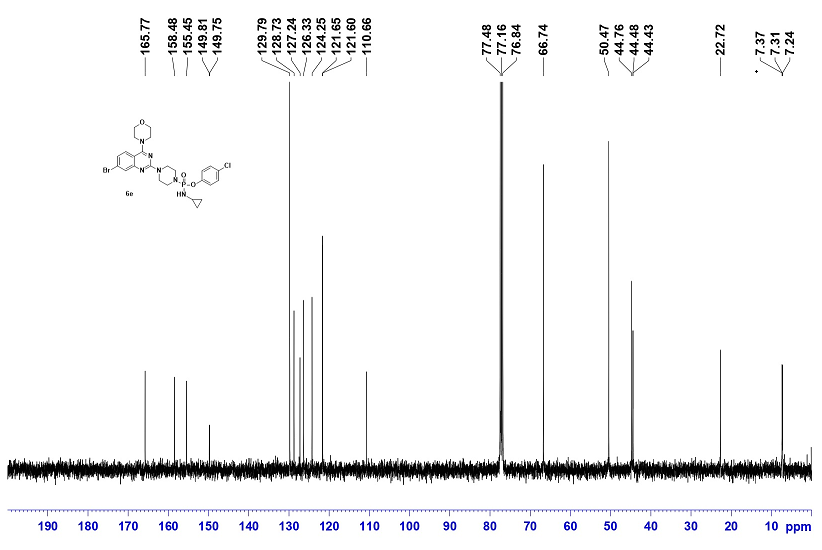


**Figure S26.** ^13^C-NMR Spectrum of Compound **6e**


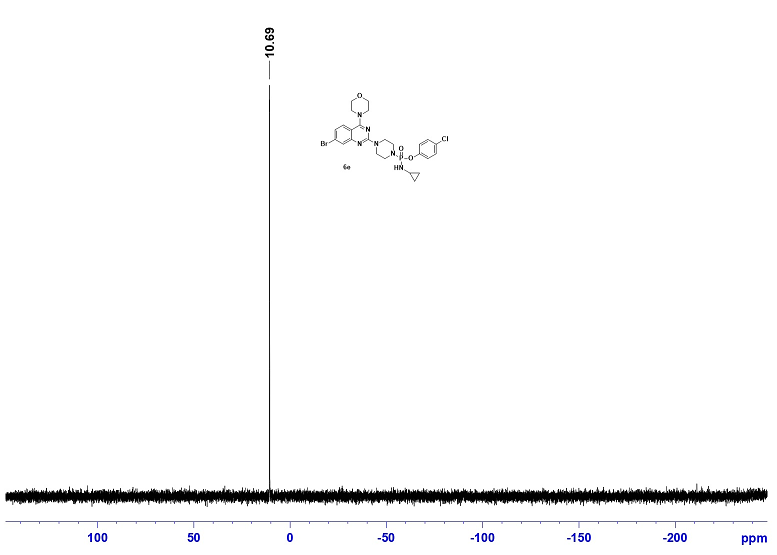


**Figure S27.** ^31^P-NMR Spectrum of Compound **6e**


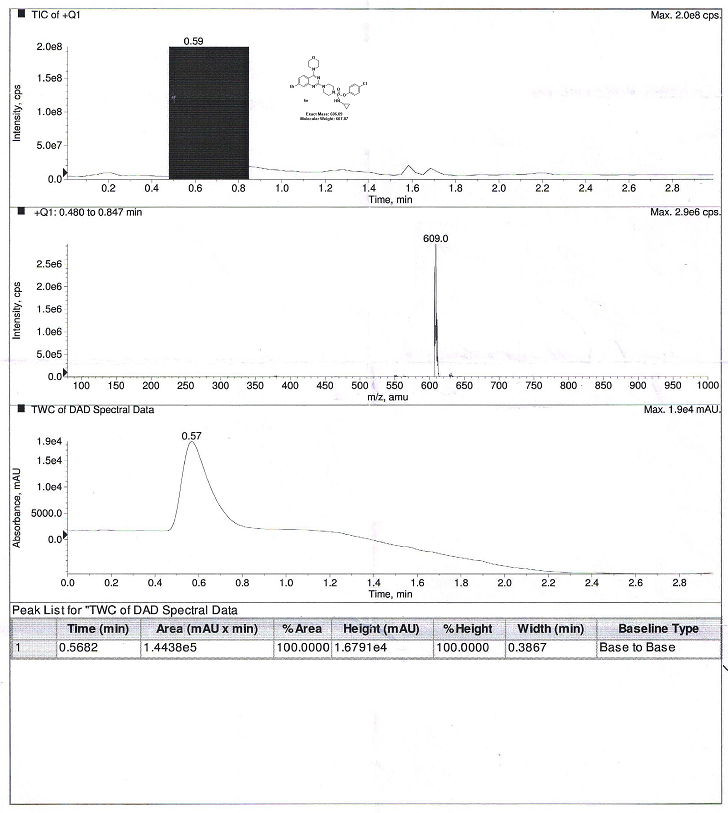


**Figure S28.** LC-MS Spectrum of Compound **6e**


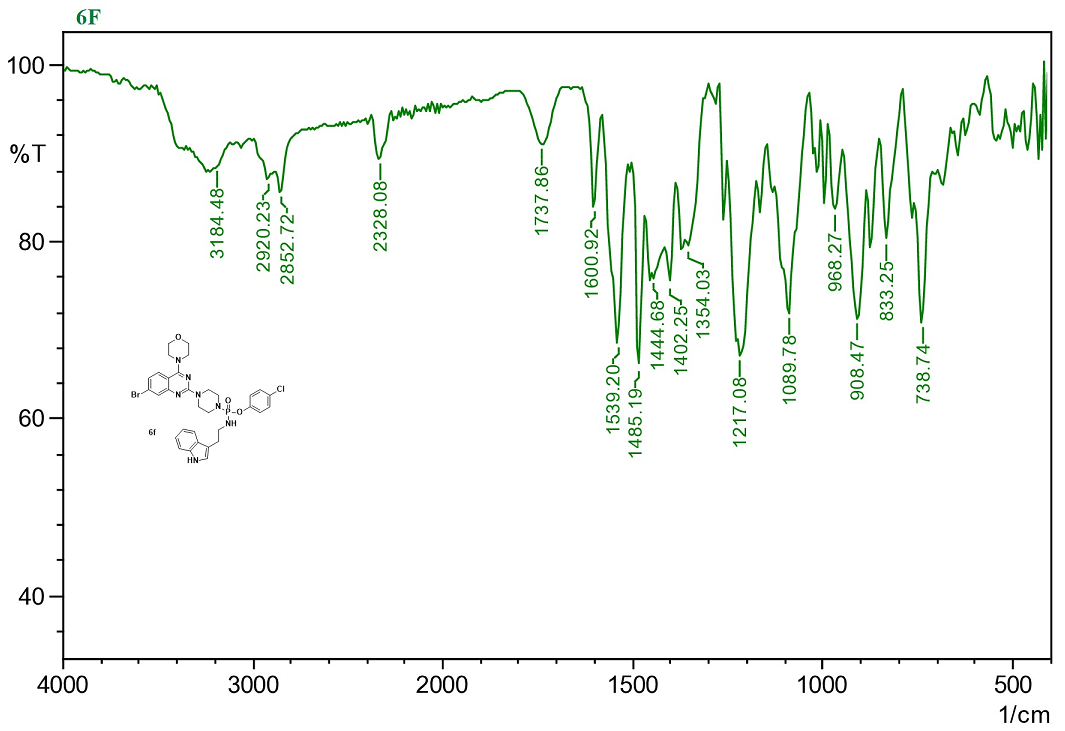


**Figure S29.** FT**-**IR Spectrum of Compound **6f**


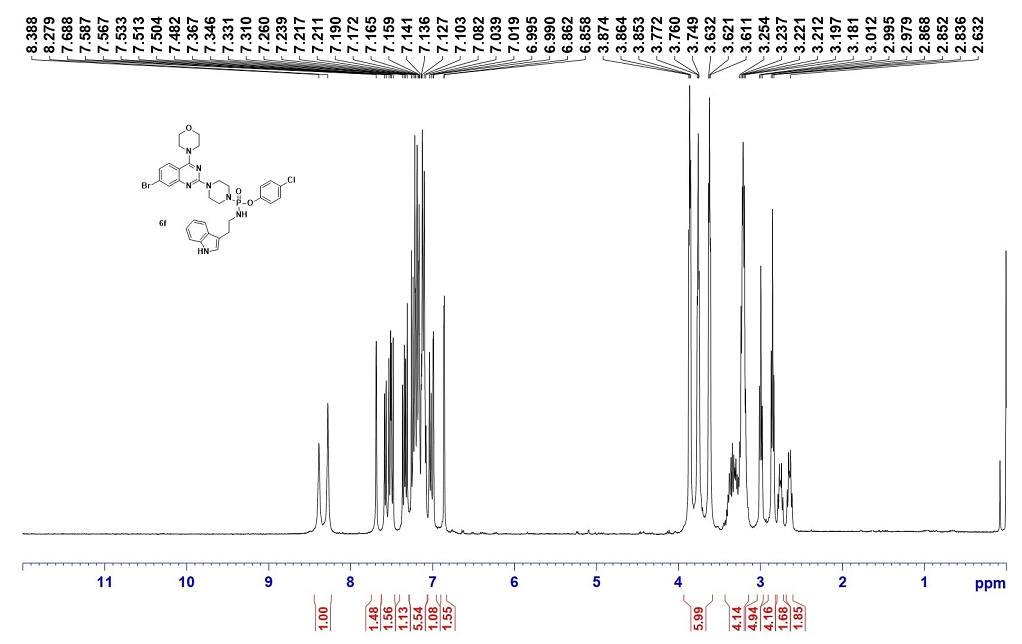


**Figure S30.** ^1^H-NMR Spectrum of Compound **6f**


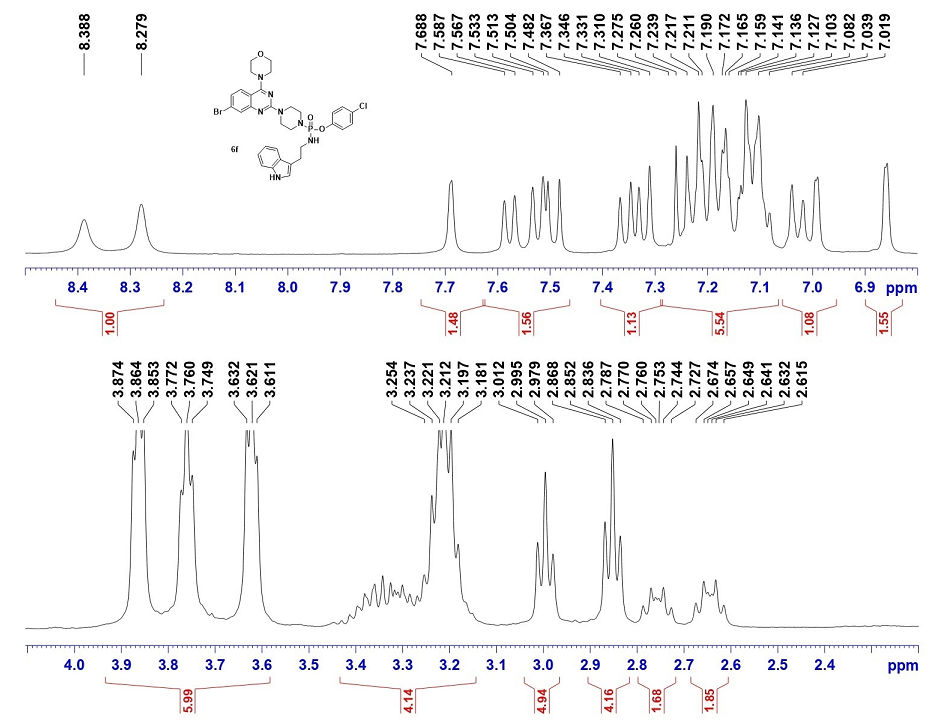


**Figure S31.** ^1^H-NMR Spectrum of Compound **6f**


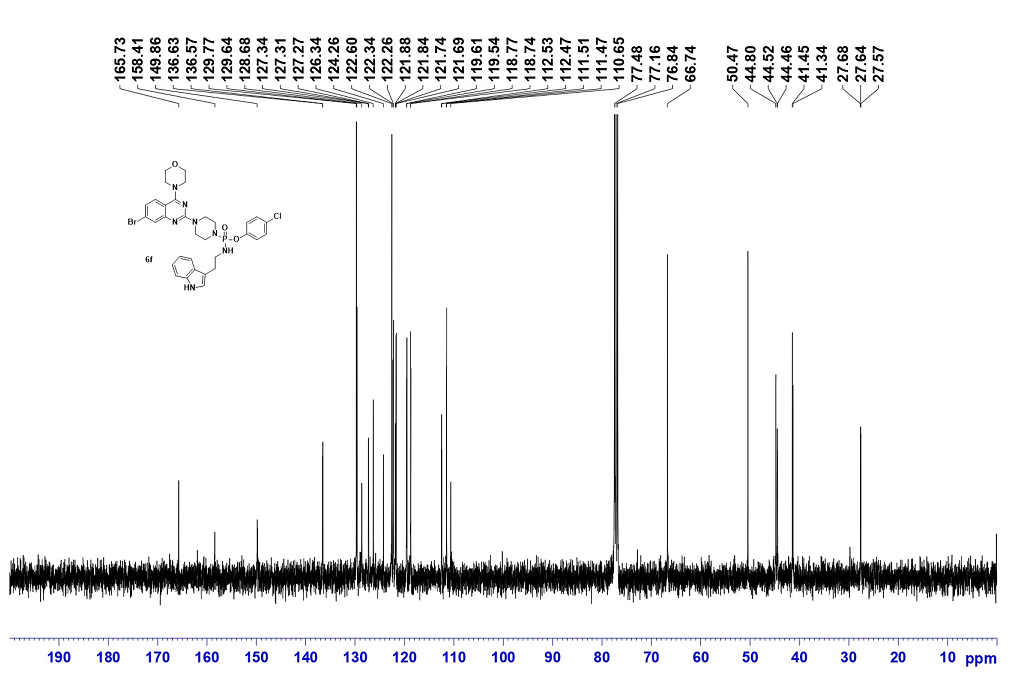


**Figure S32.** ^13^C-NMR Spectrum of Compound **6f**


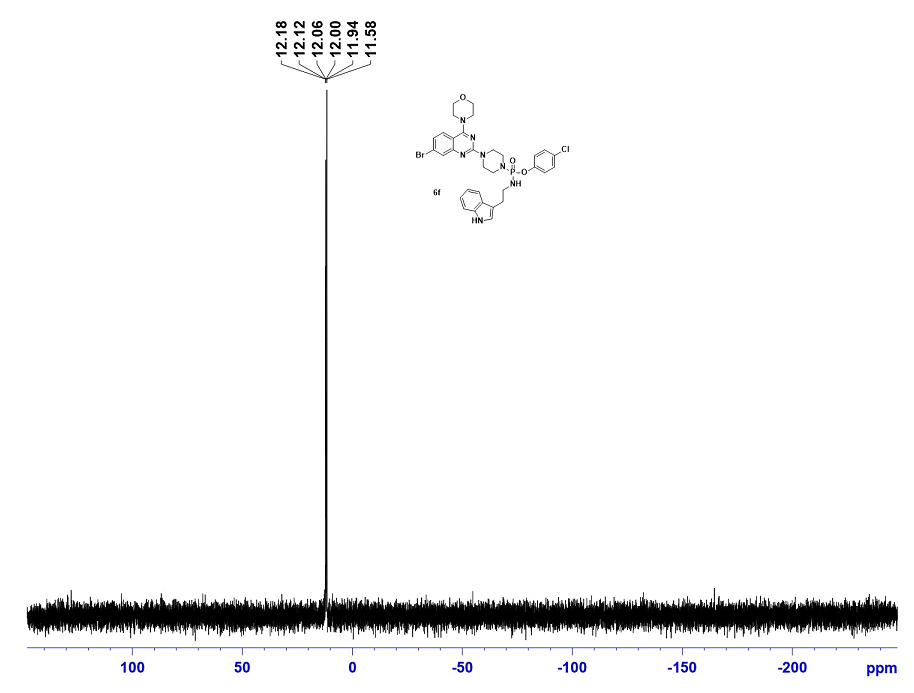


**Figure S33.** ^31^P-NMR Spectrum of Compound **6f**


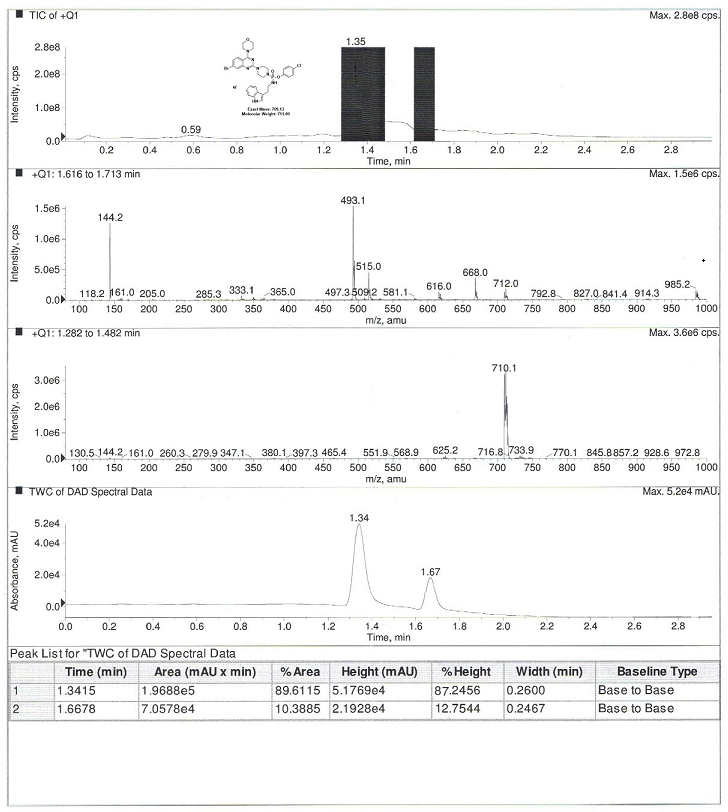


**Figure S34.** LC-MS Spectrum of Compound **6f**


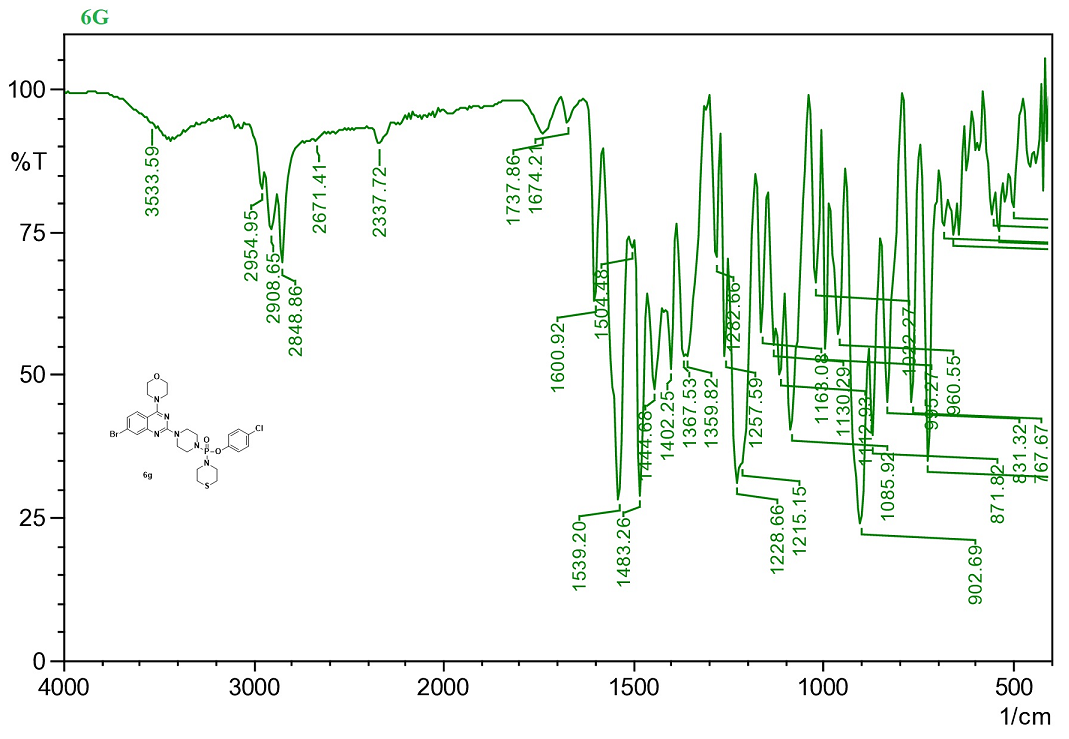


**Figure S35.** FT**-**IR Spectrum of Compound **6g**


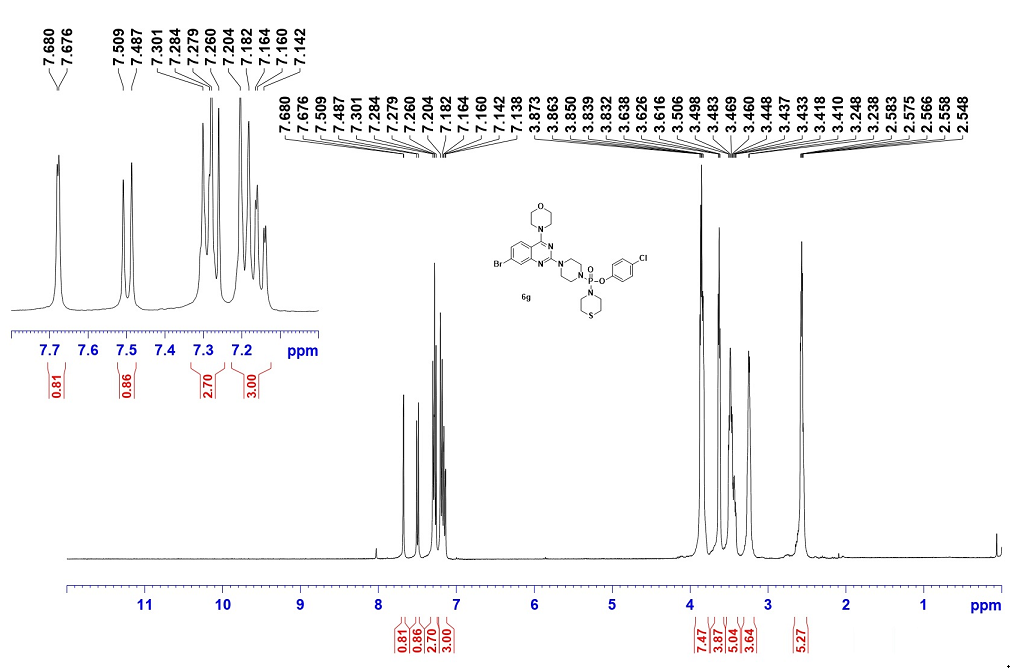


**Figure S36.** ^1^H-NMR Spectrum of Compound **6g**


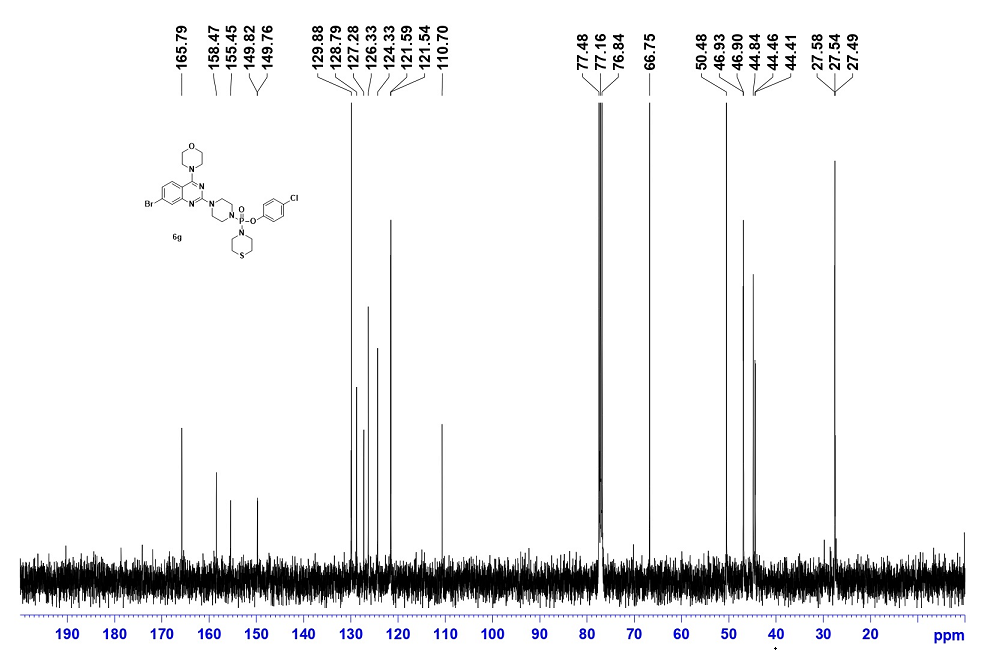


**Figure S37.** ^13^C-NMR Spectrum of Compound **6g**


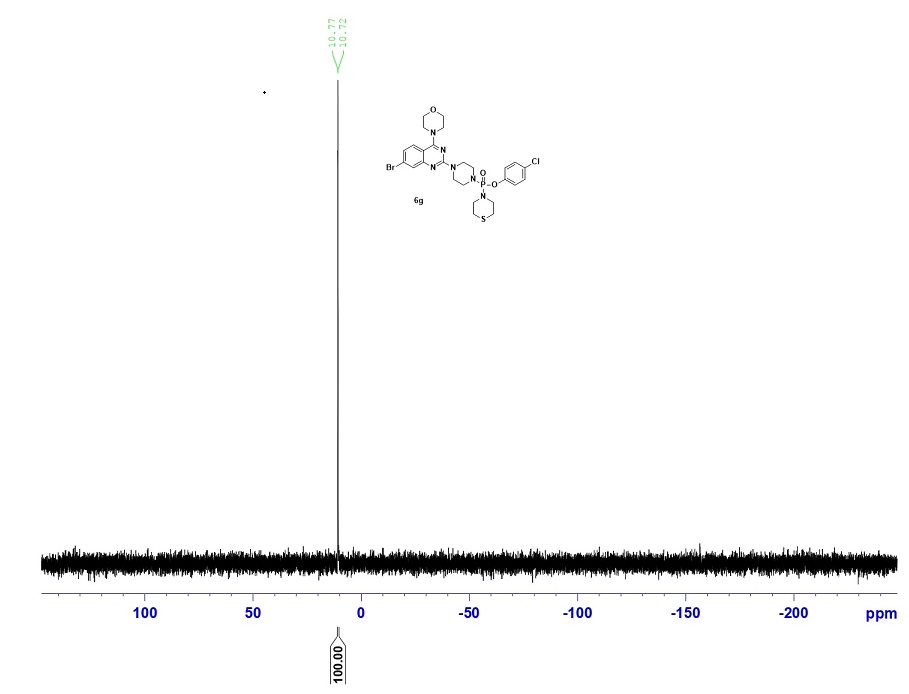


**Figure S38.** ^31^P-NMR Spectrum of Compound **6g**


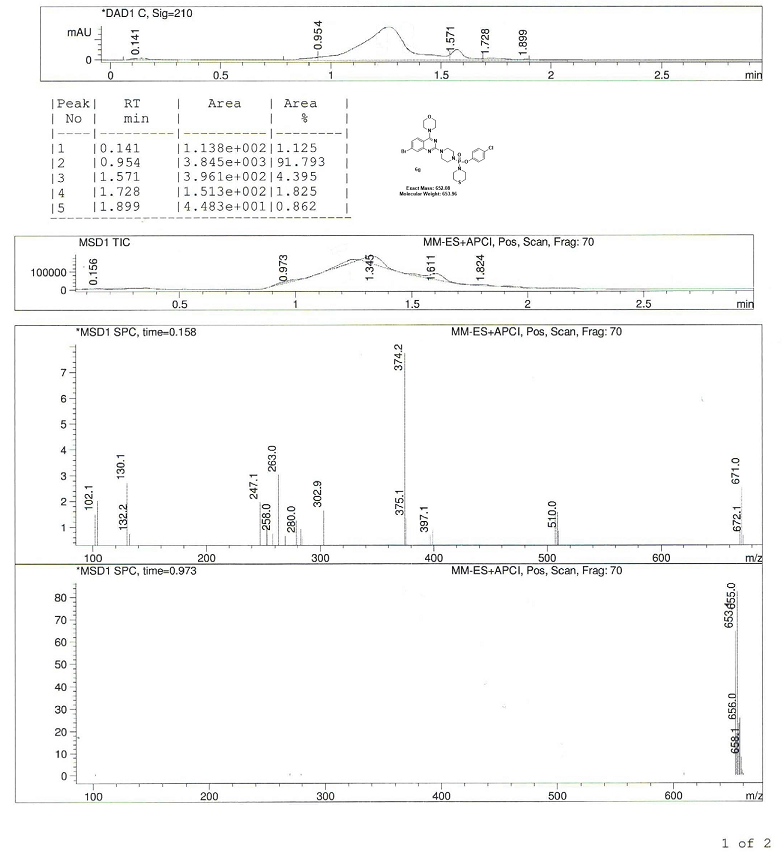

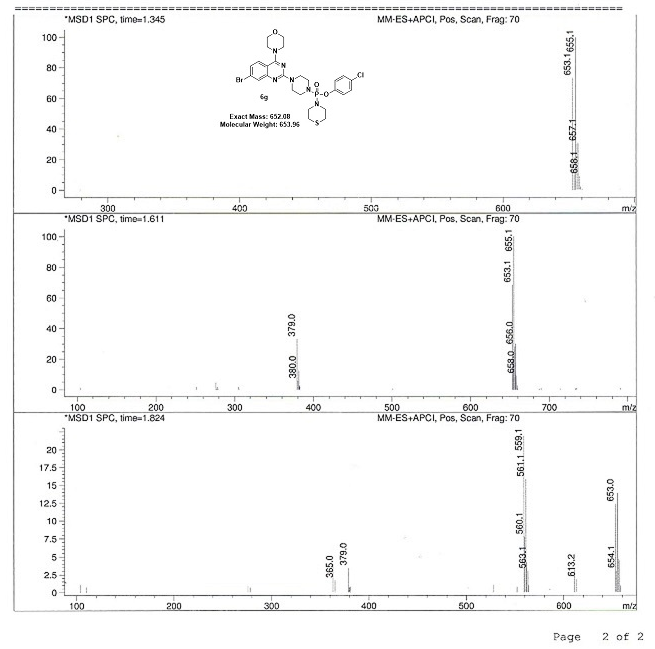


**Figure S39.** LC-MS Spectrum of Compound **6g**

**CHNS/O Elemental Analysis Data for the Compounds 6(a-g).**

**Details of CHNS/O Elemental Analysis:**

Instrument Model: Thermo Scientific Flash 2000 Organic Elemental Analyzer

Analysis Mode: CHNS/O Mode

Software: Eager Experience

**Experimental Conditions:**

Detector: Thermal Conductivity Detector

Carrier gas: He

Reference gas: He

Column: CHNS/NCS column PQS SS 2M 6X5 mm in oven at 75 °C (for CHNS)

Column: SS 1M 6X5mm (MS 5A) in oven at 100 °C (for oxygen)

Adsorption trap material: Soda lime and Anhydrone

**Program:**

CHNS furnace temp (^o^C): 950

Oxygen furnace temp (^o^C): 1060

Oven temp (^o^C): 65

Oven run time (Sec): 720 sec (for CHNS)

350 sec (for Oxygen)

Carrier gas flow (mL/min): 140

Oxygen gas flow (mL/min): 250

Reference gas flow (mL/min): 100

Sampling delay (sec): 12

Oxygen Injection end (sec): 5

Auto zero: on

Detector gain: 1

**Table S1:** CHNS/O Elemental Analysis

| S. No. | Sample Id | % of Carbon | % of Hydrogen | % of Nitrogen | % Sulphur |
| --- | --- | --- | --- | --- | --- |
| **1** | **2** | 43.61594273 | 3.286831265 | 12.56483219 | - |
| **2** | **3** | 50.64826315 | 5.258659424 | 18.32578633 | - |
| **3** | **6a** | 49.67359241 | 5.126371842 | 14.93847901 | - |
| **4** | **6b** | 48.29563247 | 4.856495217 | 13.98315742 | - |
| **5** | **6c** | 49.81255894 | 4.766891345 | 12.84524316 | - |
| **6** | **6d** | 51.39169517 | 4.416235538 | 12.27623814 | - |
| **7** | **6e** | 49.27618349 | 4.762864315 | 13.69513768 | - |
| **8** | **6f** | 53.92564833 | 4.759514836 | 13.58225741 | - |
| **9** | **6g** | 47.58366927 | 4.661934471 | 12.53852648 | 4.736271528 |
